# Integrated computational-based design of putative dual TrkA/TrkB agonists for Alzheimer’s disease: pharmacophore modelling, docking, MM/GBSA, DFT and dynamics studies

**DOI:** 10.3389/fbinf.2026.1779769

**Published:** 2026-06-01

**Authors:** A. Vignesh Pandi, Vishnu Malakar, Jeyaram Bharathi Jeyabalan, Megha Sanjai, Krishna Shevate, Kalirajan Rajagopal, B. R. Prashantha Kumar, Antony Justin

**Affiliations:** 1 Centre for Neuropharmacology and Experimental Neuroscience, Department of Pharmacology, JSS Academy of Higher Education and Research, JSS College of Pharmacy, Ooty, Tamil Nadu, India; 2 Department of Pharmacognosy, JSS Academy of Higher Education and Research, JSS College of Pharmacy, Ooty, Tamil Nadu, India; 3 Department of Pharmaceutical Chemistry, JSS Academy of Higher Education and Research, JSS College of Pharmacy, Ooty, Tamil Nadu, India; 4 Department of Pharmaceutical Chemistry, JSS Academy of Higher Education and Research, JSS College of Pharmacy, Mysuru, Karnataka, India

**Keywords:** DFT analysis, molecular dynamics, pharmacophore modelling, TrkB homology modelling, drug design

## Abstract

**Background:**

The rapid progression of Alzheimer’s disease (AD) is primarily caused by compromised neurotrophin functions and decreased tropomyosin receptor kinase expression in the basal forebrain area. The two main pathogenic features of AD are cholinergic-dependent cognitive dysfunctions and amyloidogenic-induced neurodegeneration. Concurrent stimulation of major neurotrophin signalling pathways, such as tropomyosin receptor kinases receptor A and B (TrkA and TrkB), may reduce amyloid-β-mediated neurotoxicity and cholinergic denervation in the basal forebrain, improving cognitive performance and re-establishing neuronal communication. The development of new medications with dual agonist action towards TrkA and B receptors holds enormous therapeutic potential for managing the symptoms of neurodegenerative diseases.

**Aim:**

This study aims to develop novel dual TrkA/TrkB receptor agonists for the treatment of AD by enhancing neurotrophin signalling, reducing cholinergic denervation, and mitigating amyloid-β-induced neurotoxicity.

**Methods:**

An *in silico* drug discovery pipeline was employed, involving homology and pharmacophore modelling of amitriptyline, virtual screening of ChEMBL compounds, molecular docking, ADMET, MM/GBSA analysis, DFT calculations and molecular dynamics (MD) simulations for 100 and 300 ns to assess ligand stability and binding behaviour of the ligand–protein complexes.

**Results:**

Six novel optimised quinoline analogues (OP-1 to OP-6) were identified as computationally predicted dual TrkA/TrkB agonists by molecular docking (−8.90 to −5.07 kcal/mol), MM/GBSA (−40.47 to −30.71 kcal/mol), ADMET and DFT analysis. Furthermore, OP-1, OP-2, and OP-3 exhibit stable binding interactions over 300 ns of MD simulations. The optimised compounds demonstrated favorable computational binding profiles, predicted pharmacokinetic properties, and stable receptor–ligand interactions, identifying them as promising candidates for further experimental validation as potential dual TrkA/TrkB modulators in Alzheimer’s disease.

## 1 Introduction

Neurotrophic tyrosine kinase receptors, also known as tropomyosin receptor kinases (Trk), are essential for neural development and function because they transmit neurotrophin-mediated molecular signals that control synaptic plasticity, axonal growth, and neuronal differentiation (Jeyaram Bharathi et al., 2023). The NTRK1, NTRK2, and NTRK3 genes, particularly reside on chromosomes 1q21-q22, 9q22.1, and 15q25, respectively, encompass the TrkA, TrkB, and TrkC receptors (Windisch et al., 1995a). Each receptor features different regions, including extracellular, intracellular, and transmembrane segments containing the tyrosine protein kinase domain. The extracellular domain (ECD) includes a cysteine-rich protein cluster (C1), three leucine-rich repeats (LRR), cysteine-rich repeats, and two immunoglobulin-like domains (Ig1 and Ig2) (Triaca et al., 2020; Weier et al., 1995). The LRR residues are unique to Trk receptors among tyrosine kinase receptors. Inside the cell, the domain contains five active tyrosine kinase residues: three located within the tyrosine loop, which serve as phosphorylation sites for enzymes and cytoplasmic adaptors (Cocco et al., 2018). TrkA is located on the plasma membrane, and the binding of nerve growth factor (NGF) induces dimerization within the extracellular microenvironment (Reichardt, 2006). Molecular signalling begins beyond the plasma membrane within the cytoplasm, involving pathways such as phospholipase-C-G (PLC-G), mitogen-activated protein kinase (MAPK), and phosphatidylinositol-3-kinase (PI3K). The TrkA/NGF complex is internalized through clathrin- or pincher-mediated endocytosis and micropinocytosis (Cunningham and Greene, 1998; Marlin and Li, 2015). Endocytosis can be initiated at the axonal terminal. Additionally, the signalling within endosomes, which contain the TrkA/NGF complex, may diminish interactions with the axon (Heerssen et al., 2004). Brain-derived neurotrophic factor (BDNF) is a neurotrophin that supports neuronal survival, differentiation, and maturation, while tropomyosin receptor kinase B (TrkB) serves as its principal signalling receptor.

Recent evidence suggests that alterations in BDNF downregulate BDNF transcription in the AD brain (Sampaio et al., 2017; Peng et al., 2009). By enhancing neurotrophic receptor-mediated neuronal survival, differentiation, and downstream signalling pathways, low-molecular-weight neurotrophin analogues and their engineered peptides that mimic the functional domains of neurotrophic receptors demonstrate their capacity to decrease neurotrophic effects. Peptides derived from the LRR region of TrkA and TrkB specifically bind to their respective receptors, blocking NGF and BDNF binding (Windisch et al., 1995a). Likewise, peptides engineered from NGF loops 1 and 4 bind to the IG2 domain of TrkA [3]. These findings suggest that both the LRR and Ig-like domains collaboratively facilitate high-affinity and specific neurotrophin binding (Ninkina et al., 1997; Windisch et al., 1995b). Amitriptyline serves as a dual agonist of TrkA and TrkB receptors domains (Jang et al., 2009). This is mainly because it binds to the LRR domain of TrkA, which then encourages heterodimerization and activates TrkB. Additionally, kainic acid inhibits neuronal death via a TrkA-dependent biochemical mechanism. As a result, it acts as an agonist for both TrkA and TrkB receptors and exhibits significant neurotrophic effects (Schrödinger, 2025a). These dual activation signalling cascades have potential in the regulation of neurotrophic signalling pathways and modulating neurodegenerative conditions. Despite the therapeutic promises, there is a notable lack of *in silico* and *in-vivo* studies that evaluate amitriptyline’s dual agonistic molecular effects.

Amitriptyline pharmacophore modelling highlights important physicochemical characteristics necessary for dual agonist activity and provides crucial molecular insights into the functional moiety responsible for receptor activation. The selectivity of LRR domains of TrkA and TrkB as molecular targets is required to evaluate potential agonistic actions. In this study, LRR domains were targeted for compound screening that activate either TrkA or TrkB, or other receptor heterodimerization, and act as dual agonists. Furthermore, molecular docking, MD simulations, and DFT analyses were employed to evaluate ligand–receptor interactions, conformational stability, and electronic properties of the designed compounds. While prior computational studies have targeted Trk receptors, most have focused on single-receptor screening using conventional docking approaches (Mukhtar et al., 2023). This study advances the field by applying a rigorously validated multi-tier computational framework to evaluate predicted dual TrkA/TrkB binding within the LRR domains, supported by cross-docking, long-timescale MD, free energy benchmarking, and electronic configuration analysis.

This study has aimed to screen a virtual library of small molecules that mimic the neurotrophin effects and bind to the active site of neurotrophin, in turn to stimulate a neuroprotective molecular signalling cascade. The present study focuses on computationally predict and optimize novel dual TrkA/TrkB agonists by employing pharmacophore-based virtual screening, followed by ADMET evaluation, molecular docking, and MM/GBSA analysis to assess drug-likeness and binding affinity. Selected leads are further validated through MD simulations and DFT studies and subsequently optimized to design quinoline-based analogues with enhanced dual-agonistic features.

## 2 Materials and methods

### 2.1 Pharmacophore modelling

**FIGURE 1:**
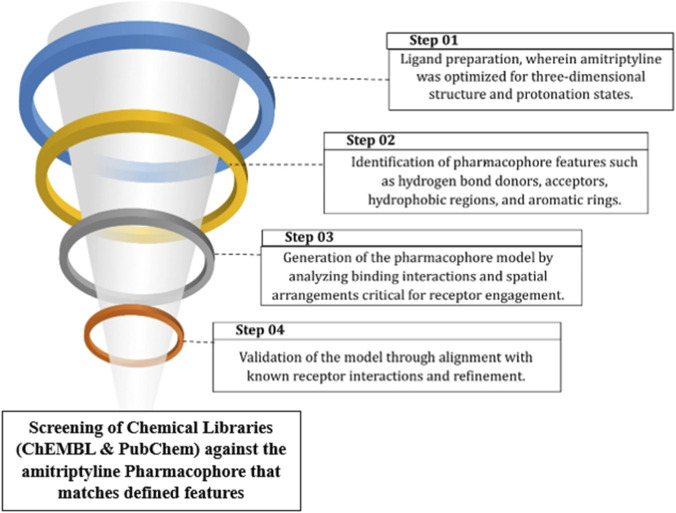
Workflow for the development of pharmacophore modelling, where amitriptyline is first prepared and optimized, followed by identification of key pharmacophoric features, model generation, and subsequent validation. The gray cone represents the sequential computational progression across these steps. Chemical libraries from PubChem and ChEMBL are then screened against the validated pharmacophore, leading to the identification of compounds that match the defined features.

Receptor ligand-based pharmacophore model was designed using amitriptyline as the reference compound, developed with the Schrödinger Suite for the progress of a Pharmacophore hypothesis. The amitriptyline structure CID: 2,160 was retrieved from the PubChem database (https://pubchem.ncbi.nlm.nih.gov/#query=amitriptyline). The optimized pharmacophore hypothesis identified four essential features for their dual agonistic effects. A single-ligand receptor pharmacophore model for amitriptyline was generated using the Develop Pharmacophore Hypotheses tool (Ligand-Based Virtual Screening module, Schrödinger Suite). The protonated form of amitriptyline at physiological pH (7.4) was prepared, energy-minimized, and conformers were generated (≤150 within a 10 kcal/mol^-1^ window). The model was built in single-ligand mode, and key features selected included two aromatic/hydrophobic centers from the tricyclic scaffold and a positively ionizable feature representing the tertiary amine, with tolerance radii of 1.2–1.5 Å. Excluded volumes were added to mimic the steric constraints of the binding pocket. The final pharmacophore hypothesis was saved and validated against a small active/decoy set before being employed as a query for virtual screening. Based on these features, compounds were screened from the ChEMBL and PubChem database comprising 185,000 small molecules. The complete workflow of the pharmacophore model is depicted in Figure 1. Compounds matching the above pharmacophoric features, with an appropriate spatial orientation and chemical properties similar to those of the amitriptyline model, were shortlisted for further analysis (Sanapalli et al., 2021; Schrödinger, 2025b).

### 2.2 Absorption, distribution, metabolism and excretion (ADME) prediction

The screened compounds were assessed for their drug-likeness properties using the QikProp module of the Schrödinger suite. ADME evaluation was calculated based on Lipinski’s Rule of Five, which features critical drug selectivity parameters, including molecular weight, partition coefficient, number of hydrogen bond donors and acceptors, along with additional permeability checkpoints, such as QPP Cacao and CNS permeability. Thus, compounds with favorable ADME predicted profiles were retained for further assessment (RCSB, 2025).

### 2.3 Target selection and protein preparation - TrkA

The ECD of the TrkA receptor was sourced from the RCSB database (PDB ID: 2IFG) (Supplementary Figure S1) (Enkavi et al., 2024), which includes the critical NGF-binding active region. The structure underwent preparation and refinement by deleting water molecules, extra protein chain, and heteroatoms, utilising Schrödinger Suite’s Protein Preparation Wizard tool (2024–25), which ensures accurate geometries and reliable torsions. The crystal structure of TrkA was processed using the Protein Preparation Wizard, applying the OPLS4 force field. Protonation states were assigned using Epik at pH 7.0 ± 2.0, missing side chains and residues were completed using Prime, and hydrogen atoms were added accordingly. The structure was then subjected to restrained energy minimization with a convergence cutoff of 0.30 Å RMSD to relieve steric clashes and optimize geometry. For molecular docking, the receptor grid was generated by centring the grid on the co-crystallized ligand binding region of TrkA. The inner grid box was defined as 10 × 10 × 10 Å and the outer grid box as 20 × 20 × 20 Å to adequately encompass the active site residues (72–97), responsible for activation of dual agonist by TrkB heterodimerization with the binding of amitriptyline, and it is crucial for optimal ligand binding. The van der Waals scaling factor was maintained at 1.0 with a partial charge cutoff of 0.25. No positional or hydrogen-bond constraints were applied during grid generation. The receptor grid for TrkA (X: 44.43, Y: 26.01, Z: 39.68) was focused on the first LRR domain (residues 72–97).

### 2.4 Homology modelling and validation - TrkB

The TrkB homology model was constructed in BioLuminate Schrodinger Suite, utilizing closely related protein templates, due to the lack of a full-resolution TrkB ECD structure, especially the Lucine-rich domain (Rojas, 2025). Hence, during modelling, basic parameters were selected for specific active leucine-rich motif (LRM) residues, spanning from 92 to 137 within the ECD of TrkB, as this region was the primary target for further investigation. The TrkB target–template alignment was manually reviewed and adjusted as necessary to ensure the correct matching of functionally important residues. For regions with low homology, a multiple sequence alignment approach was used to improve alignment accuracy. Prime generated the 3D model using the alignment: structurally conserved regions were copied from the template backbone, while insertions/deletions were modelled as loops. Loop regions were modelled using Prime’s knowledge-based loop search and, where appropriate, *ab initio* sampling. Side-chain conformations across the model (including remodeled loops and any mutated residues) were assigned from rotamer libraries and optimized for low energy (Schrö dinger, 2025).

**FIGURE 2:**
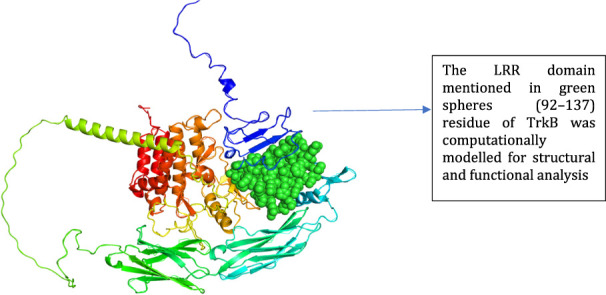
Prepared structure of TrkB homology model protein.

The initial model was processed through the Protein Preparation Wizard to correct protonation states, add missing atoms and hydrogens, fix bond orders, and remove obvious steric overlaps. Energy minimization was performed using the OPLS4 force field to relieve clashes and optimize geometry. Model quality was assessed with Prime’s Protein Reliability Report (Supplementary Table S1) and by Ramachandran plot analysis of backbone φ/ψ angles (Supplementary Figure S2). The final model exhibited the majority of residues in favored or allowed regions and an acceptable reliability score for downstream modelling, as shown in Supplementary Figure S1 and Supplementary Table S1. Therefore, the grid box created in the LRM (X: 18.78, Y: 0.22, Z: 26.86) spans residues 92–137 in the TrkB ECD. Maintaining structural integrity and facilitating particular chemical recognition are important functions of this area, which is identified as critical for ligand binding sites. The refined TrkB homology model is therefore suitable for subsequent structure-based studies. Homology model of TrkB with grid box generated on LRM is portrayed in Figure 2.

### 2.5 Molecular docking and MMGBSA assay

The screened compounds were docked in the active site of the TrkA and TrkB LRR domains to study the possible residue-ligand interactions and binding affinity using the Glide module of Schrodinger Suite. The LigPrep module of the Schrodinger suite was utilized for refinement of the screened compounds, which included geometry optimization, assigning of protonation states at default EPIK settings at pH 7 ± 2 with the OPLS4 force field. The docking was conducted using three precision modes: HTVS, SP, and XP based on the binding affinity. Further, the docking validation was performed by redocking to the TrkA and TrkB domains (RMSD ≤2.0 Å). Top hits were selected for having substantially better docking scores and key interactions, compared to the standard amitriptyline. Glide docked postures were used as input for prime MM/GBSA (Molecular Mechanics/Generalized Born Surface Area) computations. Protein-ligand complex binding free energies (ΔGbind) were estimated using the VSGB 2.1 implicit solvent model and the OPLS4 force field. The resulting output files, including energy values summarized in a. csv format, were analyzed (Schrödinger, 2025c).

### 2.6 Density functional theory (DFT) calculations

The Jaguar module of the Schrödinger Suite was used to perform DFT calculations on the lead compounds that made the short list to obtain information about their electrical characteristics. The energy gaps associated with border in the frontier molecular orbitals including HOMO and LUMO were calculated to identify electronic configuration, chemical stability, and reactivity. The molecular structure was prepared in Maestro either by building or importing the ligand, followed by geometry optimisation using DFT (B3LYP or equivalent functional with 6-31G (d, p) basis set) to obtain a stable low-energy conformation. A single-point energy calculation was then performed on the optimised structure using the same functional and basis set to generate orbital data. HOMO and LUMO molecular orbitals were visualised through the Surfaces and Contours panel, and their corresponding energy values were obtained. The HOMO–LUMO energy gap was calculated, with the gap size interpreted in terms of molecular reactivity, electronic hardness/softness, and possible electronic transitions relevant to UV-Visible absorption properties (Schrödinger, 2025d).

### 2.7 MD simulations

The stability of the docked complex was assessed through the MD simulation using the Desmond module of the Schrödinger suite. The SPC water model was used to solvate the docked complex after it was positioned in an orthorhombic box at a buffer distance of 10 Å. Counter ions and 0.15 M NaCl were added to neutralise the system further. The solvated system was minimized and equilibrated using the default protocol featuring the NPT ensemble throughout the simulation using the OPLS4 force field, and the Nosè–Hoover chain thermostat and Martyna–Tobiase–Klein barostat, respectively, maintained the temperature of 300 K and the pressure of 1 atm at interval times of 2 ps and 1 ps. Thus, the docked complex was simulated under the predefined biological conditions for 100–300 ns and evaluated using RMSD, P-L interaction timeline, and P-L interaction histogram (Schrödinger, 2025e).

### 2.8 Scaffold refinement and molecular design

The Ligand Designer module was used to further optimise the lead molecule, producing a library of new quinoline analogues (Schrödinger, 2025f). The 3D structure of the target protein was analyzed to identify the binding pocket, key residues, and interaction patterns from bound ligands. Property maps were generated using polar and hydrophobic probes to highlight favorable regions for hydrogen bond donors, acceptors, and hydrophobic contacts. Guided by these maps, ligand design and optimization were performed in Ligand Designer through iterative growth, bio-isostere replacement, isostere scanning, and cyclisation strategies. Designed ligands were refined to establish favorable protein–ligand interactions, while the multi-receptor mode was applied to optimize selectivity and minimize off-target binding. Top candidates underwent molecular docking, ADME profiling, and over 300 ns MD simulations to confirm protein-ligand complex stability and interaction profiles for potential drug development. This streamlined workflow integrated advanced computational platforms to identify and optimize dual TrkA/TrkB agonists, ensuring rigorous pre-selection of candidates for downstream experimental validation.

## 3 Results

### 3.1 Pharmacophore modelling: ligand-based drug design

**FIGURE 3:**
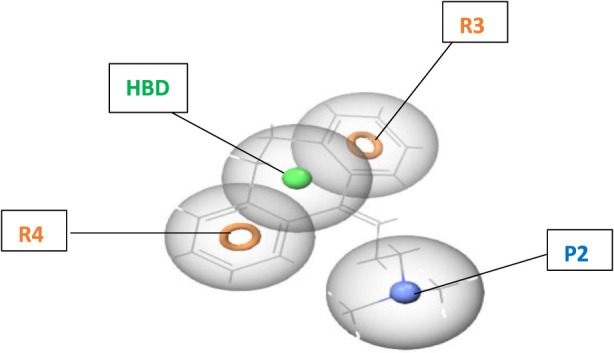
Pharmacophore for amitriptyline comprises one hydrogen bond donor (HBD), two aromatic rings (R3, R4), and one positive ionizable feature (P2).

One hydrogen bond donor (HBD), two aromatic rings (R3, R4), and one positive ionisable feature (P2) made up the hypothesis that was assessed via receptor ligand-based pharmacophore model in Schrödinger Suite 2024–25 version (Figure 3) using statistical parameters including fitness score, matched sites, Phase score, Victor score, and volume score, followed by virtual screening of ∼185,000 compounds from ChEMBL and PubChem, based on small molecules 1,000 were screened which yielded 500 hits among that 100 hits were scrutinized based on statistical parameter, with the top five summarized in Table 1. Validation was performed using known tricyclic antidepressants (e.g., nortriptyline, imipramine, clomipramine) and presumed inactive, as well as a decoy set from DUD-E (Directory of Useful Decoys – Enhanced). The pharmacophore retrieved actives with higher scores than inactive and showed significant enrichment, as confirmed by enrichment factor (EF) and goodness-of-fit (GH) score. ROC analysis yielded an AUC >0.80, demonstrating strong discriminative ability depicted in the Supplementary Tables S2-S5. Collectively, these results confirm that the amitriptyline-based pharmacophore is robust, predictive, and suitable for virtual screening to identify novel scaffolds. The structure-based pharmacophore validation was performed using the receptor–ligand complex of amitriptyline depicted in (Supplementary Figure S3). The generated model successfully reproduced the key pharmacophoric features observed in the ligand-based hypothesis, specifically R3 and R4 (aromatic ring features), HBD (hydrogen bond donor), and P2 (hydrophobic feature) corresponding to amitriptyline. The spatial arrangement and inter-feature distances were preserved, confirming structural consistency with the original single-reference pharmacophore model. Hypothesis validation demonstrated that the compound satisfied 4 out of 5 pharmacophoric features with optimal geometric alignment, indicating good internal consistency. Importantly, the validated structure-based model yielded feature mapping and energetic contributions comparable to the original amitriptyline-derived hypothesis, supporting that the refined model captures the essential interaction pattern associated with its reported dual TrkA/TrkB binding profile. These findings confirm that the pharmacophore hypothesis is not arbitrary but structurally supported by receptor–ligand interaction geometry represented in Supplementary Table S6.

**TABLE 1:** Top 5 compounds matching the amitriptyline pharmacophore hypothesis.

Compound ID	Fitness score	Matched sites	Phase screen score	Victor score	Volume score
Amitriptyline (standard)	1.976	4	1.976	0.831	0.618
Cmpd-1	1.942	4	1.942	0.812	0.602
Cmpd-2	1.910	4	1.910	0.804	0.590
Cmpd-3	1.885	3	1.885	0.798	0.584
Cmpd-4	1.872	3	1.872	0.789	0.573
Cmpd-5	1.861	3	1.861	0.781	0.569

### 3.2 ADME screening

For additional docking analysis, 500 molecules in total were selected. Lipinski’s Rule of Five, a commonly used metric for oral drug-likeness prediction, served as the basis for the filtering criteria. These indicators are essential for assessing a molecule’s capacity to display ideal ADME characteristics in the human body. This methodical process guarantees that a sizable molecular dataset is refined into a high-quality subset of drug-like compounds that are prepared for subsequent docking investigations and possible lead identification for additional structure-based drug design. All screened compounds exhibited consistent ADME profiles, with 100% predicted human oral absorption and no violations of Lipinski’s rule of five. The only exception was Chembl51183, which showed slightly lower oral absorption (88%). In contrast, the standard compound exhibited one Lipinski rule violation. Overall, the results indicate favorable drug-likeness and oral bioavailability for the screened compounds. Table 2 summarises the ADME analysis results for the top hits.

**TABLE 2:** ADME analysis of top 8 lead molecules.

ChEMBL ID	MW	HBD	HBA	QPlog Po/w	QPPCaco	% human oral absorption	Rule of five	CNS
Chembl35741	324.22	2	4	8.1	2,663.30	100	0	1
Chembl4065673	288.32	1	1	4.95	6,803.01	100	0	1
Chembl4864026	286.33	2	2	4.00	2,351.02	100	0	0
Chembl49609	271.32	1	3	3.82	3,353.76	100	0	0
Chembl48152	286.33	3	2	3.92	2036.95	100	0	0
Chembl51183	269.34	2	5	2.13	559.66	88	0	1
Chembl105882	286.33	2	2	3.91	1982.21	100	0	0
Chembl48715	302.33	3	3	3.33	1,270.79	100	0	1
Standard	277.40	0	2	5.03	2,470.58	100	1	1

Reference Range of MW: 130–725 Da; <500 preferred. HBD: 0–5, HBA: 2–10 (≤12 tolerated), QPlog, Po/w: 2.0 to +6.5 (0–5 optimal), QPP, Caco (nm/sec): <25 poor, >500 excellent (>100 good), % Human Oral Absorption: <25% poor, >80% high, Rule of Five: ≤1 violation (MW ≤ 500, HBD ≤5, HBA ≤10, logP ≤5), CNS, index: 2 (inactive) to +2 (active).

### 3.3 Molecular docking

In the TrkA LRR domain, the ligand (Chembl35741) is anchored through strong hydrogen bonding interactions of its–NH and–OH groups with *Asp86*, while a cluster of surrounding hydrophobic residues (*Leu56, Ile98, Pro63*) helps stabilize the aromatic scaffold and fluorinated substituents. Conversely, in the TrkB LRR domain, the ligand Chembl35741 engages in hydrogen bonding between its–OH group and *Glu59*, along with an additional hydrogen bond or electrostatic interaction between–NH group and *Arg57*. Chembl4864026 interacts with TrkA mainly through hydrogen bonds with *His 60* and *Gly 89*, along with hydrophobic contacts with several leucine and other residues. In contrast, its binding to TrkB is characterised by a key hydrogen bond with *Cys32* and additional interactions with nearby amino acids such as *Arg27, Ala28*, and *Phe30*. These findings shown distinct receptor-specific binding profiles that may help optimize selectivity for this ligand. Chembl51183 primarily binds to TrkA via a hydrogen bond with *Asp86* and interacts with *His60, Pro63, Glu66*, and several leucine rich residues. While TrkB forms hydrogen bonds with *Asp86, Leu87, and Arg88*, complemented by hydrophobic contacts with nearby residues. These patterns reveal unique binding modes with key target-specific contacts for each receptor. Amitriptyline, used as a standard drug, mainly binds to TrkB through a hydrogen bond with *Asp47* and interacts with residues such as *Pro33, Cys32, Phe30, Arg27, Trp 26*, as well as several polar contacts including *Ser35, Thr34, Lys37*, and *Cys36, 38*. In the TrkA receptor, amitriptyline strongly interacts with hydrogen bond such as *Asp86*, and also contacts *Glu66, Pro63, His60, Leu56, Arg88*, and glycine residues, which stabilise its binding. These patterns highlight key target-specific contacts for amitriptyline with both TrkA and TrkB.

Amitriptyline binds to the TrkA receptor with a higher affinity (−3 kcal/mol). The compounds were methodically assessed using their amino acid interaction patterns and docking score (Kim et al., 2024). Encouraging receptor heterodimerization, this guarantees its capacity as a dual function agonist. It's interesting to note that in docking studies, the screened compounds outperformed the typical ones, as demonstrated in Supplementary Tables S7, S8. A comparison has been made between the main amino acid residue bindings of amitriptyline and the interactions of the screened compounds (Supplementary Figure S4). The highest binding affinity and persistent interactions shown towards the TrkA receptor’s LRR domain served as the foundation for compound Chembl35741. Additionally, to validate the docking studies, Protein and docked complex superimposition was carried out, and the deviation was found below 3 Å, suggesting site-specific docking (Supplementary Figure S5).

### 3.4 Molecular dynamics analysis

The ECD of TrkA has been selected for additional MD simulations in this investigation. Since 2IFG possesses the ECD structure for TrkA, which contains the LRR domain, it was chosen based on its distinctive structural characteristics.

Initially, MD simulations were performed for amitriptyline targeting the leucine-rich domain of TrkA. During these simulations, the structural dynamics of the protein were largely determined by its initial compactness and intrinsic rigidity. The protein root-mean-square deviation (RMSD) between 30 and 50 ns proteins showed a maximum deviation of 14 Å, which then stabilised to around 10 Å after 70 ns. The average ligand RMSD was found to be 7.0 Å. The stability was observed at 6 Å after 70 ns. The high RMSD indicates more deviation of Amitriptyline within the binding pocket. The presence of consistent interactions, including hydrophobic contacts, ionic interactions, hydrogen bonds, and water bridges, highlights robust and sustained binding. The detailed illustration of RMSD and Protein ligand contacts is shown in Supplementary Figure S6.

#### 3.4.1 Molecular dynamics of Chembl35741 against TrkA LRR domain of 2IFG

The protein RMSD (Cα atoms) is represented by the blue line, which first rises in the early stages of the simulation before stabilising around 3.0–3.5 Å after about 25–30 nsec. This shows that the protein achieves equilibrium and keeps a rather stable shape for the duration of the simulation. The ligand RMSD (Lig fit Prot), represented by the red line, has greater variations between about 3 and 7, especially between 20 and 60 nsec. The variations become less noticeable after about 60 nsec, suggesting better stability. The RMSD values stay within a tolerable range, indicating that the ligand stays inside the binding site without considerable displacement or unbinding, despite the ligand exhibiting more dynamic behaviour than the protein. The contact map offers comprehensive information about ligand interactions specific to residues. The heatmap illustrates the frequency of connections between the ligand and particular residues, whereas the top plot displays the total number of contacts over time. Interestingly, *Asp57, His60, Arg85*, and *Asp86* show long-lasting interactions with the ligand, indicating that they play important roles in keeping the ligand stable in the binding site. The strength and frequency of interactions are shown by the colour gradient’s intensity, where darker hues imply residues that create four or more connections (Supplementary Figure S7). Displays a detailed representation of RMSD and protein ligand interactions.

#### 3.4.2 Molecular dynamics of CHEMBL4864026 against TrkA LRR domain of 2IFG

The RMSD of the protein backbone (Cα atoms) after 100 ns of MD simulation is shown by the blue line. It rises for the first 15 ns or so, then fluctuates steadily between 6.5 and 9.5 Å, with sporadic maxima above 11 Å. For big proteins, where flexible loop sections and numerous domains can contribute to larger global RMSD values, these relatively high values are regarded as acceptable. Crucially, the protein enters a conformational equilibrium when the trajectory stabilises without continuing to rise. The ligand RMSD (Lig fit Prot) is represented by the red line, which stabilises after the first 20–25 ns and exhibits fewer oscillations, mostly between 2.5 and 6. This implies that during the simulation, the ligand stays in a comparatively steady location within the binding pocket. The ligand does not seem to dissolve despite the moderate kinetics, suggesting sustained binding and compatibility with the binding site. Throughout the 100 ns simulation, the protein-ligand contact profile stays constant, supporting the notion of stable interaction. Important residues involved in sustained binding are also shown by the heatmap. The darker orange to red patches indicates that *Leu71, Leu87, Gly89, Leu90, Gly91, and Leu93* in particular exhibit frequent and durable interactions (>4 contacts). These residues probably contribute significantly to the ligand’s stability in the binding pocket, indicating a stable and advantageous binding mode over time (Supplementary Figure S8). provides a detailed representation of RMSD and protein ligand interactions.

#### 3.4.3 Molecular dynamics of CHEMBL51183 against TrkA LRR domain of 2IFG

The protein RMSD (Cα atoms) is represented by the blue line, which first increases throughout the simulation’s early stages before stabilising at 5–8 Å between 20 and 70 nsec with sporadic variations. The protein RMSD exhibits more noticeable fluctuation after around 70 nsec, peaking about 10–11 Å, which might suggest some structural rearrangements or flexibility, particularly in terminal or loop areas. These RMSD values are regarded as appropriate and do not indicate unravelling or instability due to the size of the protein. The red line, representing the ligand RMSD (Lig fit Prot), remains relatively stable throughout the simulation with values generally between 2 Å and 6 Å during the first 70 nsec. After this time, though, the RMSD rises to approximately 12–14 Å, indicating an increase in volatility. Although it appears to completely detach, this indicates a potential change in ligand orientation or partial relocation within the binding pocket. In the final part of the trajectory, the ligand shows increased mobility while still being attached to the protein. Key residues *Leu87, Arg85, Gly91, and Asp57* participating in hydrophobic, hydrogen bonding, and water bridge interactions are highlighted in the bar graph; *Arg85* additionally displays ionic connections. Persistent interactions with *Asp57, His60, Arg85, Asp86, Leu87, and Gly91* during the 100 ns simulation are shown in the heatmap. The steady contact plot verifies constant ligand engagement throughout, while darker shades show robust, persistent binding (≥4 contacts). RMSD, RMSF, and protein ligand interactions are illustrated in detail in Supplementary Figure S9.

### 3.5 Identification of novel compounds by optimization of Chembl35741

**FIGURE 4:**
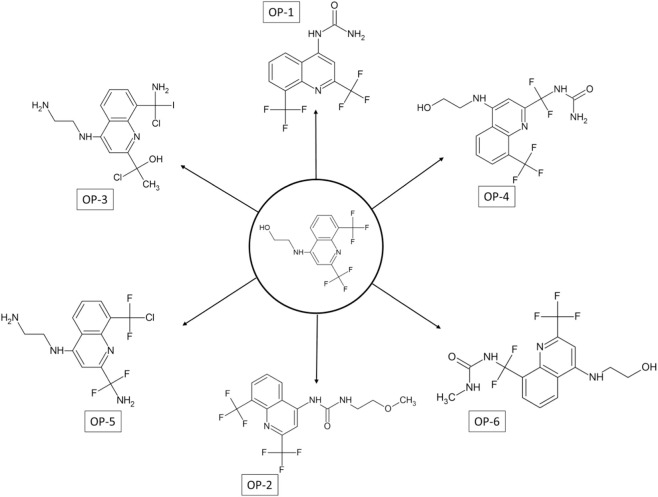
Library of newly designed quinoline analogues derived from the optimisation of Chembl35741.

Based on strong molecular docking, high stability in MD simulations (Supplementary Table S9), and an ideal HOMO-LUMO gap (Supplementary Table S10), Chembl35741 was found to be a viable scaffold for TrkA receptor activation. Using these characteristics, Chembl35741 was optimised using the Schrödinger Suite’s Ligand Designer module, creating a library of new quinoline analogues, as shown in (Figure 4). Improved docking scores and advantageous ADMET profiles led to the selection of the top 6 compounds. The molecule with the best combination of docking, physicochemical, and ADMET properties was chosen for more in-depth research from this optimised subset. The prolonged simulation duration validated the compound’s promise as a lead candidate for additional preclinical development and offered solid insights into the temporal stability of the interaction.

### 3.6 Molecular docking studies of designed compounds

**FIGURE 5:**
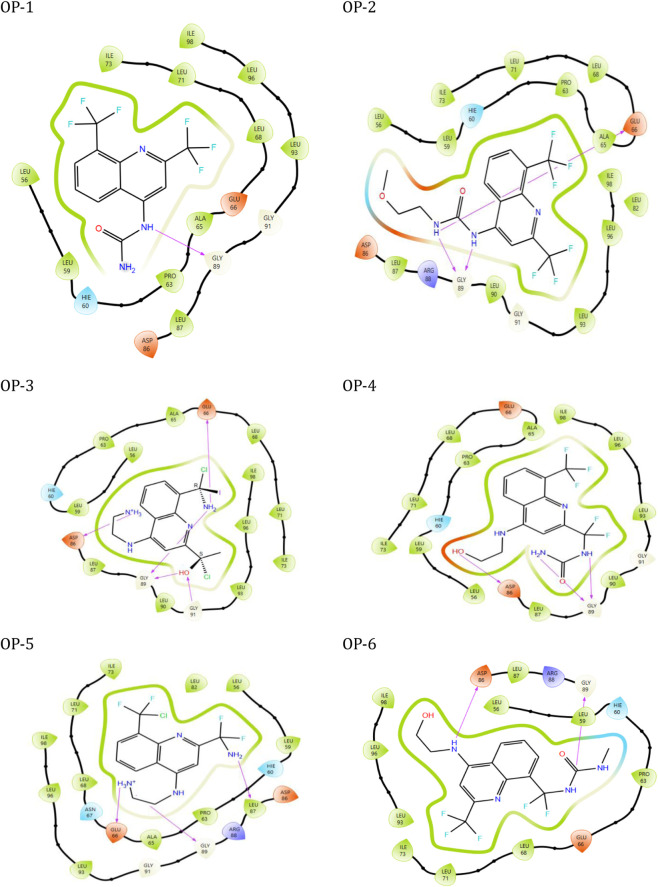
2D interaction of quinoline analogues derived from the optimised compounds (Purple arrows indicate hydrogen bonds; green shaded contour represents hydrophobic contacts; colored residue circles denote residue type (green = hydrophobic, orange = acidic, blue = basic, cyan = polar); black line represents the protein backbone).

The docking scores of all six optimized compounds (OP-1 to OP-6) against the LRR domain of the TrkA receptor were notably more negative than the standard amitriptyline, signifying a possible higher affinity of the designed compounds (Figure 5; Table 3).

**TABLE 3:** Docking scores of optimised compounds (OP-1 to OP-6).

Compound	Docking score (kcal/mol)	Amino acid interaction
OP-1	−8.447	Gly89
OP-2	−8.151	Glu66, Gly89
OP-3	−7.650	Glu66, Arg86, Gly89, Gly91
OP-4	−7.461	Asp86, Gly89
OP-5	−7.610	Glu66, Gly89, Asp86
OP-6	−5.075	Asp86, Gly89
Standard	−4.061	Asp86

OP-1 interacts with amino acids such as *Asp86* (hydrogen bonding*), Leu67 and Tyr90*, engaging in hydrophobic interactions and π–π stacking. Whereas OP-2 showed hydrogen bonding with *Asp86* similar to amitriptyline and additional contacts involving residues such as *Phe89 and Val85* through hydrophobic forces. OP-3 reported hydrogen bonding with *Asp86* and additional pi-pi stacking with *Tyr90*, mimicking the binding mode of amitriptyline. OP-4, like others, forms a hydrogen bond with *Asp86* and hydrophobic contacts with *Leu67* and other neighboring residues. OP-5 exhibits hydrogen bonding with *Asp86*, supported by π–π interactions with aromatic residues in the LRR domain. OP-6 similarly engages *Asp86* via hydrogen bonding and hydrophobic engagement with adjacent residues like *Tyr90*. Amitriptyline’s known interaction in this domain primarily involved hydrogen bonding with *Asp86*, an essential interaction that all six optimized compounds replicate, confirming a conserved binding mode. Moreover, the optimized compounds display enhanced binding scores relative to amitriptyline, indicating stronger binding interactions.

Hence, all six optimized compounds share key amino acid interactions, especially at *Asp86* through hydrogen bonding, as observed with the standard. Their superior docking scores and binding interactions suggest they are promising candidates. To ensure methodological robustness and reproducibility, multiple validation strategies were implemented. Redocking was performed to assess pose reproducibility, and since amitriptyline is not co-crystallized in the TrkA structure (PDB ID: 2IFG), self-docking of the reference binding region was conducted. The RMSD between the reference and redocked poses was calculated, with values of 1.68 Å for TrkA and 1.92 Å for the TrkB modelling, both within the acceptable threshold (≤2.0 Å), confirming docking accuracy (Supplementary Table S11). The cross-docking analysis demonstrates consistent binding modes across both TrkA and TrkB receptors, with preservation of key conserved residues, particularly *Asp86* and *Arg88*. Comparable docking scores and maintained interaction patterns support the structural plausibility of dual receptor engagement and confirm the robustness of the docking protocol (Supplementary Table S12). Enrichment validation showed good screening performance (ROC-AUC = 0.82, EF1% = 12.4, GH = 0.71) (Supplementary Table S13). Additionally, docking was performed in triplicate with a fixed random seed, yielding standard deviations below 0.5 kcal/mol, confirming computational stability and reproducibility mentioned in Supplementary Table S14.

### 3.7 MM-GBSA/binding free energy calculations of designed compounds

The MM-GBSA results for the six screened compounds reveal comprehensive insights into their binding energetics, with each energy component providing specific information about the nature of molecular interactions (Table 4). The total ΔG Bind values for all compounds range from around −30.71 to −40.47 kcal/mol, which are considered favorable, as values below −25 kcal/mol typically suggest strong and stable binding in molecular docking assessments. The ΔG Bind Coulomb values reflect electrostatic interactions and are most effective when significantly negative; here, the values span from −21.41 to −53.33 kcal/mol, with the more negative figures reflecting stronger ionic/electrostatic attraction. ΔG Bind Covalent values are zero for all compounds, indicating that no covalent bonding was predicted, which is typical and desirable for reversible drug candidates unless a covalent inhibitor is intended. The ΔG Bind H-bond scores represent the strength of hydrogen bonding and range from −0.52 to −2.32 kcal/mol; while relatively small, these values contribute favorably, typical good hydrogen bond contributions range between −1 and −3 kcal/mol. The ΔG Bind Lipo values between −9.08 and −20.39 kcal/mol demonstrate the contribution of hydrophobic forces, with more negative values indicating stronger lipophilic interactions; scores below −10 kcal/mol are considered particularly beneficial. Lastly, the ΔG Bind van der Waals (vdW) energies range from −15.46 to −38.14 kcal/mol, where negative values (especially those less than −10 kcal/mol) point to effective molecular packing and complementarity. Collectively, these readings affirm that the compounds have energetically favorable binding profiles, aligning with expected ranges for promising drug candidates compare to standard (TOXCHARITE, 2025; Ossila, 2025).

**TABLE 4:** MM-GBSA/binding free energy calculations of optimised compounds (OP1 to OP-6).

Compound	dG bind	dG bind coulomb	dG bind covalent	dG bind Hbond	dG bind Lipo	dG bind vdW
OP-1	−36.01	−20.84	4.58	−1.63	−10.45	−31.11
OP-2	−38.12	−25.24	0	−1.53	−16.46	−24.40
OP-3	−30.71	−39.59	0	−1.08	−9.08	−27.40
OP-4	−34.71	−21.41	0	−2.06	−13.16	−38.14
OP-5	−40.47	−53.33	0	−2.32	−20.39	−29.14
OP-6	−39.35	−26.40	0	−0.52	−17.06	−15.46
Standard	−13.66	−20.49	14.74	−0.11	−33.47	−7.27

### 3.8 ADME-TOX prediction of designed compounds

The ADMET and drug-likeness results for compounds OP-1 to OP-6 demonstrate several promising pharmacokinetic complying the Lipinsk’s rule of five where the compounds have molecular weights (MW) ranging from 336 to 455 Da, the number of rotatable bonds <10 suggesting favorable flexibility for absorption followed by Hydrogen bond donors (HBd) < 5 and acceptors (HBa) < 7 indicative of good oral bioavailability compared to standard (Table 5).

**TABLE 5:** ADME analysis of optimised compounds (OP1 to OP-6).

Comp ound	# rotor	MW	HBd	HBa	Qplog Po/W	QPP caco	QPP MDCK	QPlog HERG	% human oral absorption	CNS	PSA	Rule of five
OP-1	1	323	3	3	2.252	395.42	5,358	−2.982	86	1	70.00F	0
OP-2	4	374	3	7	1.984	170.79	568.9	−4.778	78	1	104.88	0
OP-3	6	375	3	5	3.182	329.33	494.5	−5.041	90	0	53.179	0
OP-4	6	364	5	6	1.351	105.65	718.6	−3.504	86	2	104.88	0
OP-5	5	455	5	5	1.660	37.542	175.6	−4.212	80	1	86.424	0
OP-6	4	378	4	6	2.148	521.84	2,684	−3.541	88	1	85.114	0
Standard	3	277	0	2	5.034	2,470	1,454	−6.092	100	1	2.689	1

Reference Range of #Rotors (Rotatable Bonds): 0–10 acceptable, MW: 130–725 Da; <500 preferred, HBD: 0–5, HBA: 2–10 (≤12 tolerated), QPlog, Po/w: 2.0 to +6.5 (0–5 optimal),QPP, Caco (nm/sec): <25 poor, >500 excellent (>100 good),QPP MDCK (nm/sec): <25 poor, >500 excellent (>100 good),QPlog HERG: > −5 acceptable, < −5 indicates potential cardiotoxicity risk, % Human Oral Absorption: <25% poor, >80% high,PSA (Polar Surface Area): <140 Å^2^ acceptable, <90 Å^2^ preferred for good oral absorption, <70 Å^2^ preferred for CNS, penetration, Rule of Five: ≤1 violation (MW ≤ 500, HBD ≤5, HBA ≤10, logP ≤5), CNS, index: 2 (inactive) to +2 (active).

QPlogPo/W (logP, octanol/water partition coefficient) values ranges were found to be 1.351 and 3.182, fitting the ideal drug-like window (usually between 1 and 3), favouring membrane permeability without excessive lipophilicity or hydrophilicity. QPP Caco-2 cell and MDCK cell permeability values, with most above 100, support potential for good intestinal absorption and blood-brain barrier penetration; higher values indicate better permeability. QPlogHERG predictions, all well >−5, suggest a low risk of cardiac toxicity due to hERG blockade. Human oral absorption percentages, mostly in the 67%–90% range, are considered satisfactory, indicating likely efficient uptake upon administration. CNS activity prediction is low (0 or 1), aligning with either low or moderate central nervous system penetration, which may be desirable depending on the intended indication. The polar surface area (PSA) values, mostly <140 < 140 Å^2^, support good permeability and oral absorption. All compounds comply with the Rule of Five, reinforcing drug-likeness.

The toxicity prediction analysis for the six top-screened compounds performed in ProTox 3.0 indicated low overall risk for major organ-specific toxicities (Table 6). For all compounds, there were no predicted signs of hepatotoxicity, neurotoxicity, respiratory toxicity, or cardiotoxicity except nephrotoxicity, suggesting a favorable safety profile with respect to the main physiological systems (Google, 2025). The “Predicted Toxicity” score, ranging from 4 to 5 across compounds, falls within a low-risk category commonly used in computational toxicity assessment, reinforcing their potential suitability for further development and *in-vivo* testing. This early toxicity screening supports prioritizing these molecules for more advanced pharmacological investigations and experimental validation. Overall, these metrics collectively demonstrate that each compound possesses favorable ADMET characteristics compared to standard, sufficient to advance them in further preclinical investigations.

**TABLE 6:** Toxicity analysis of optimised compounds (OP1 to OP6).

Compound	Hepatotoxicity	Neurotoxicity	Nephrotoxicity	Respiratory toxicity	Cardio toxicity	Predicted toxicity
OP-1	-	-	+	-	-	4
OP-2	-	-	+	-	-	5
OP-3	-	-	+	-	-	4
OP-4	-	-	+	-	-	4
OP-5	-	-	+	-	-	5
OP-6	-	-	+	-	-	4
Standard	-	-	-	-	+	4

Active= (+), Inactive= (−), (1) Extremely Toxicity (2) Highly Toxic, (3) Moderately Toxic, (4) slightly toxic, (5) Low Toxicity.

### 3.9 Scoring function and MM/GBSA binding energy evaluation

Docking poses were initially ranked using the GlideScore scoring function (kcal/mol), which integrates empirical terms accounting for hydrogen bonding, hydrophobic interactions, van der Waals contacts, electrostatic contributions, lipophilic enclosure, and desolvation penalties. The top-ranked poses from Glide XP docking were visually inspected to ensure proper orientation within the binding pocket and meaningful interactions with key active-site residues. To further validate binding affinity and reduce false-positive predictions inherent to docking-only approaches, Prime MM/GBSA calculations were performed on the selected protein–ligand complexes. These calculations employed the VSGB 2.1 implicit solvent model in combination with the OPLS4 force field, enabling a more physics-based estimation of binding free energy by incorporating molecular mechanics energies, solvation effects, and surface area contributions. For each complex, the binding free energy (ΔG_bind) was calculated using the standard thermodynamic cycle by subtracting the sum of the free energies of the unbound protein and ligand from the free energy of the protein–ligand complex, thereby estimating the energetic favorability of binding. Compounds exhibiting ΔG_bind < −25 kcal/mol were considered to demonstrate significant binding affinity, consistent with commonly accepted thresholds for stable protein–ligand interactions in virtual screening studies. The integration of GlideScore for rapid empirical ranking followed by MM/GBSA refinement provided a hierarchical and energetically rigorous compound prioritization strategy, thereby improving predictive reliability and minimizing overestimation of docking scores alone.

### 3.10 Molecular dynamics of designed quinoline analogue against TrkA LRR binding site of 2IFG

**FIGURE 6:**
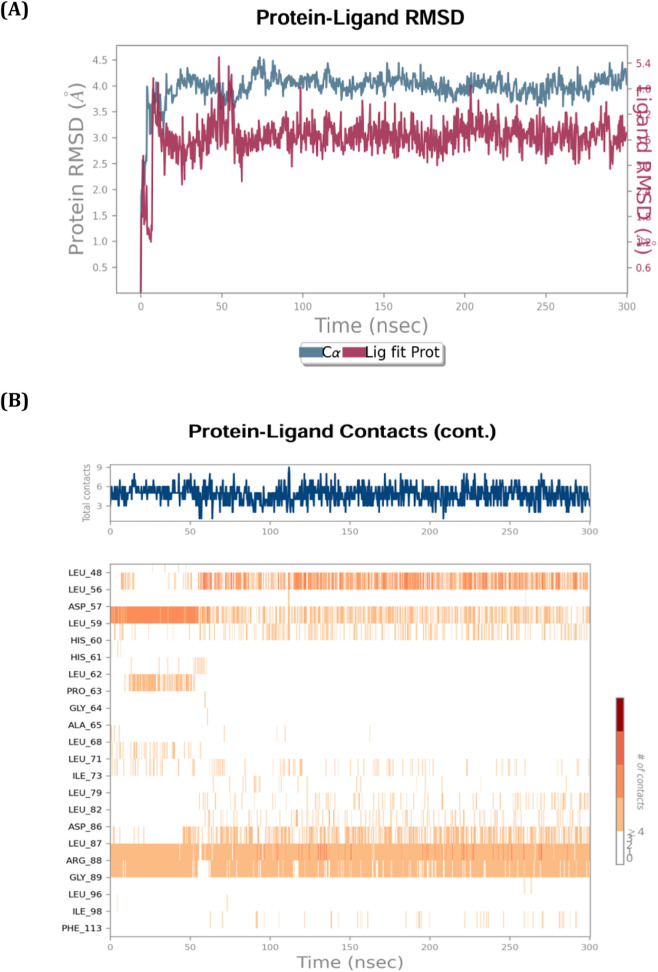
Molecular dynamics of quinoline analogue (OP-1) against the TrkA LRR domain binding site: RMSD **(A)** and Protein-Ligand Interaction **(B)** over 300 ns.

**FIGURE 7:**
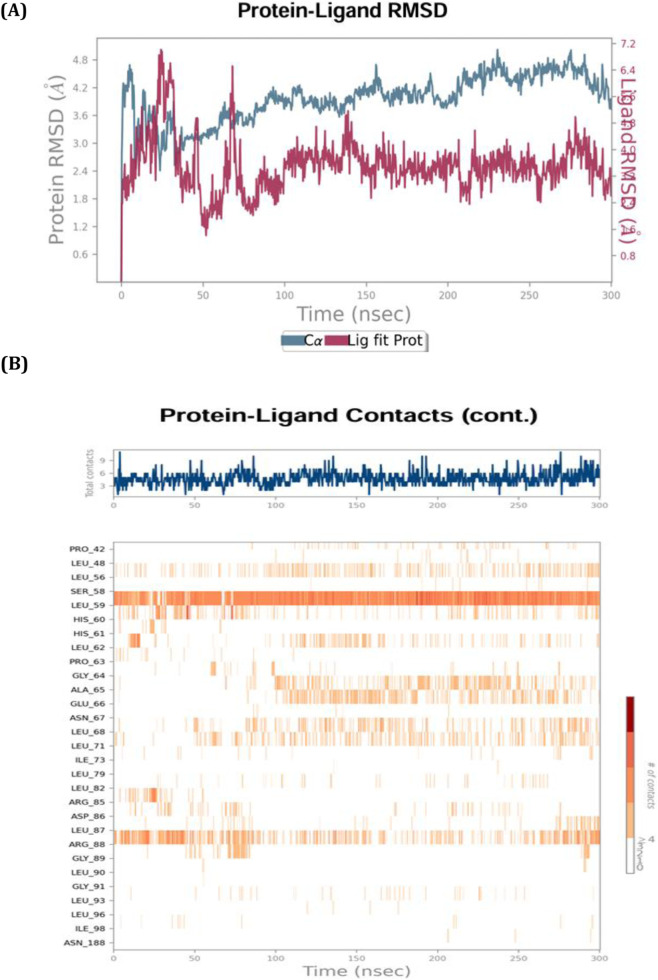
Molecular dynamics of quinoline analogue (OP-2) against the TrkA LRR domain binding site: RMSD **(A)** and Protein-Ligand Interaction **(B)** over 300 ns.

**FIGURE 8:**
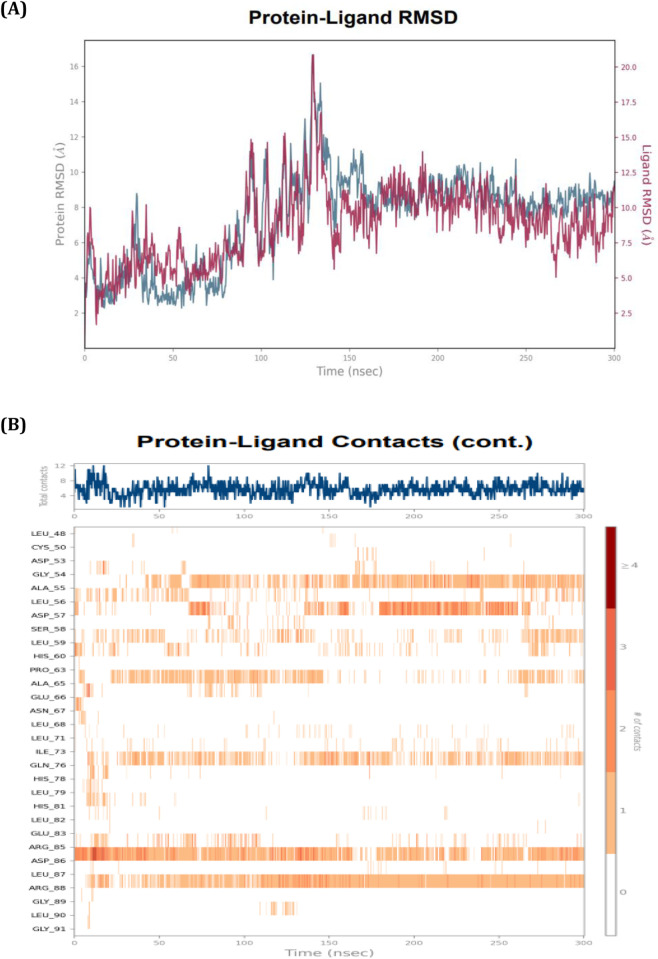
Molecular dynamics of the quinoline analogue (OP-3) against the TrkA LRR domain: binding site RMSD **(A)** and Protein-Ligand Interaction **(B)** over 300 ns.

The 300 ns MD simulation demonstrated stable binding of the quinoline analogue OP-1 to TrkA (Figure 6). Protein backbone RMSD stabilized at ∼3.8–4.2 Å after equilibration, consistent with the dynamic nature of large, active receptors and indicating conformational equilibrium without drift. Ligand RMSD showed limited fluctuations following initial equilibration, confirming sustained ligand retention within the binding pocket throughout the simulation. Protein–ligand contact analysis revealed persistent interactions with key residues, including hydrophobic contacts with *Ala55, Ala65*, and *Leu59*, strong salt bridges with *Asp53, Asp57,* and *Asp86*, hydrogen bonding (including water-mediated) with *Gln76*, and a prominent electrostatic interaction with *Arg88.* Together, these results confirm the dynamic stability and favorable binding of OP-1 to TrkA over the 300 ns trajectory. MD analysis over 300 ns demonstrated that the OP-2 remained stably bound to TrkA (Figure 7). After an initial adjustment phase, the protein backbone RMSD plateaued at ∼4.0–4.8 Å, consistent with the flexible nature of large receptors and indicative of a well-equilibrated system. The ligand RMSD similarly stabilised following early fluctuations, confirming persistent binding within the active site. Protein–ligand interaction profiling revealed long-lasting contacts with key residues, particularly hydrophobic interactions with *Leu59, Leu68*, and *Leu87*, along with stable electrostatic interactions involving *Asp86* and *Arg88*. Additional transient hydrogen-bonding interactions contributed to sustained ligand engagement. Collectively, these findings support the structural stability and favourable binding behaviour of the ligand–TrkA complex throughout the simulation. Over the course of 300 ns, this MD simulation of the OP-3 complex with the big protein TrkA, shown in (Figure 8), displays the predicted high backbone RMSD typical of large, active proteins, with overall stabilisation indicative of a thoroughly equilibrated system. Crucially, as the simulation continues, the trajectory stabilises without any continuous upward drift, indicating that the protein has reached a conformational equilibrium. Contained, stable binding is confirmed by the ligand RMSD, which closely monitors protein mobility. After the first part of the trajectory, the ligand RMSD stabilises and shows smaller, more controlled variations, primarily between 2.5 and 20Å. Throughout the 300 ns simulation, this pattern indicates a constant ligand position in relation to the protein, suggesting continuous binding within the pocket. Although there is no indication of ligand separation, moderate movements are seen, suggesting compatibility and long-term engagement within the binding site. Strong and persistent ligand binding to important protein residues, such as *Ala55, Asp57*, and *Arg86*, is revealed by protein-ligand contact analysis through hydrophobic, ionic, hydrogen bond, and water bridge interactions and the ligand binds stably through key salt bridges with *Asp53, Asp57*, and *Asp86*, hydrogen bonding (including water-mediated) with *Gln76*, and a strong electrostatic interaction with *Arg88*, supported by hydrophobic contacts with *Ala55, Ala65*, and *Leu59*, indicating favorable and stable binding revealed in ligand -protein contacts during the course of the 300 ns simulation. The dynamic stability, biological plausibility, and dependability of the molecular simulation for large protein-ligand systems are validated by these results. Binding energies were benchmarked against the reference ligand amitriptyline to provide comparative affinity context. MM/GBSA energies were averaged over frames extracted from the final 50 ns of 300 ns MD simulations, with standard deviations reported to account for trajectory variability. RMSD, hydrogen bond occupancy, and stability metrics were analyzed to ensure robust binding energy estimation and statistical reliability, in which all prioritised compounds exhibited binding energies comparable to or stronger than amitriptyline, supporting their predicted binding affinity (Supplementary Table S15). Low standard deviations (<2 kcal/mol) indicate stable energy convergence, while RMSD values below 2.5 Å and consistent hydrogen bond occupancy confirm structural stability and sustained receptor interaction throughout the simulation as shown in Supplementary Table S16.

RMSD and RMSF analyses were quantified by reporting mean values and standard deviations across the equilibrated trajectory segment (Supplementary Table S17). Convergence was evaluated by monitoring RMSD stabilization and comparing early (0–100 ns), middle (100–200 ns), and late (200–300 ns) simulation intervals, confirming structural equilibration after ∼50 n. Replicate simulations were performed to validate trajectory reproducibility, and averaged stability metrics were calculated (Supplementary Table S18). The low RMSD variability and consistent RMSF profiles across replicates confirm structural stability and dynamic reliability of the protein–ligand complexes were all complexes exhibited stable RMSD values below 2.5 Å with low standard deviation, indicating structural stability. Convergence was achieved within ∼50–65 ns, and consistent radius of gyration values confirm maintenance of protein compactness. Minimal inter-run deviation (<0.05 Å) confirms strong reproducibility and trajectory robustness across independent simulations. To further validate the stability of the systems, total energy analysis was performed, which showed stable energy convergence throughout the simulation. RMSD and RMSF analyses were compared with the reference drug Amitriptyline, revealing that OP-1 and OP-2 exhibit superior stability and reduced flexibility, supporting their potential as promising candidates depicted in Supplementary Figures S10, S11.

### 3.11 DFT calculations

**FIGURE 9:**
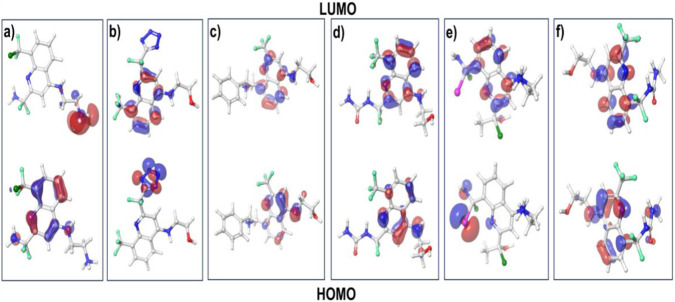
Frontier molecular orbital (FMO) representation of optimised compounds; **(a)** OP-1, **(b)** OP-2, **(c)** OP-3, **(d)** OP-4, **(e)** OP-5, and **(f)** OP-6.

The frontier molecular orbital representations (Figure 9) illustrate graphical representations of the spatial distribution of the electron density in the HOMO and LUMO of designed compounds (Lane et al., 2018). DFT calculations were performed on the designed compounds (OP-1 to OP-6) to determine their theoretical reactivity and possible biological activity as TrkA/TrkB dual agonists. Frontier molecular orbital FMO analysis, including HOMO and LUMO, gives vital information on electron-donating and electron-withdrawing features of the compounds, which is vital in determining how they will interact with their biological targets. Table 7 represents calculated HOMO and LUMO values, along with the corresponding energy gap and related quantum chemical descriptors, including chemical hardness (η) and chemical softness (S).

**TABLE 7:** DFT calculations of optimised compounds (OP-1 to OP-6).

Compound	HOMO (eV)	LUMO (eV)	Energy gap (eV)	Chemical hardness (η)	Chemical softness (S)
OP-1	−8.898	−4.898	4	2	0.5
OP-2	−2.531	0.952	3.483	1.742	0.574
OP-3	−8.626	−4.544	4.082	2.041	0.49
OP-4	−6.204	−1.850	4.354	2.177	0.459
OP-5	−8.544	−5.034	3.510	1.755	0.57
OP-6	−6.150	−1.796	4.354	2.177	0.459

The HOMO values were found in the range of - 8.898 eV (OP-1) to - 2.531 eV (OP-2), indicating variable electron-donating capacities of the designed series. Compounds OP-1, OP-3, and OP-5 had the most negative HOMO values, indicating greater stability. On the other hand, compound OP-2 reported the negative minimum HOMO (-2.531 eV), implying a higher electron-donating potential, and this could increase the electrostatic interaction of the Compound OP-2 with the negatively charged residues in the binding sites of TrkA/TrkB domains.

The ranges of the LUMO values were found from - 5.034 eV (OP-5) to 0.952 eV (OP-2). The negative values of LUMO of most compounds (OP-1, OP-3, OP-4, OP-5, and OP-6) show that they exhibit favorable electron-accepting ability and thermodynamic stability in the ground state. Whereas compound OP-2 reported a positive LUMO value, implying a difference in the electronic properties among the designed compounds.

The values of the energy gap, were ranged from 3.510 eV (OP-5) to 4.354 eV (OP-4 and OP-6). The energy gaps greater than 3.5 eV tend to signify compounds that are stable enough kinetically and have fewer chances of spontaneous decomposition. Compounds OP-4 and OP-6 (4.354 eV and 4.354 eV, respectively) had the largest energy gaps, indicating better thermodynamic stability. Conversely, compound OP-5 had the least energy gap (3.510 eV), which suggests a relatively high electronic accessibility and, consequently, it could be more biologically reactive because of its relatively lower HOMO-LUMO gap (Cummings et al., 2021).

The chemical hardness (η) is used as a quantitative measure of the ability of a compound to resist structural deformation and charge transfer in the process of intermolecular interactions. Compounds OP-4 and OP-6 demonstrated the most chemical hardness (2.177 eV), Compound OP-5, on the other hand, reported the least chemical hardness (1.755 eV), implying it has more flexibility in the TrkA/TrkB domain.

## 4 Discussion

AD, being a multifaceted disease, single-target therapies often fail to address the interplay of Aβ pathology, tauopathy, neuroinflammation, and neuronal death. Despite extensive research for the treatment of AD over the decades, the options remain limited, and clinical outcomes are often very suboptimal (Zhang et al., 2024). Treatments available are regularly initiated after significant neuronal loss, when cognitive functions are irreversibly impaired, while the therapeutic strategies primarily target the symptoms rather than addressing the underlying neurodegenerative processes (Kazim and Iqbal, 2016). Hence, the complexities of AD pathophysiology necessitate a paradigm shift towards complete and modified treatment approaches to improve patient outcomes. These persistent failures of single-target therapies require a paradigm shift in the conceptualization of AD and its subsequent therapeutic approaches. The integration of neurotrophic signalling pathways into the understanding of AD pathogenesis offers a paradigm shift. Rather than staging AD solely through protein aggregation, investigating the dysregulation of neurotrophin-mediated survival mechanisms provides a more comprehensive framework that encompasses multiple aspects of disease progression (Gao et al., 2022). In AD, reduced TrkA expression impairs NGF signalling, promoting cholinergic degeneration, cognitive decline, neuroinflammation, and Aβ plaque formation. Similarly, decreased BDNF/TrkB signalling contributes to synaptic dysfunction, hippocampal atrophy, tau hyperphosphorylation, and heightened neuroinflammation, collectively exacerbating disease progression (Poon et al., 2013; Wang et al., 2022).

Emerging evidence indicates that Aβ oligomers can directly impair BDNF-TrkB signalling through a cascade of mechanisms, including receptor internalization, disruption of receptor trafficking, and interference with downstream signalling cascades (Lindsay, 1996). Simultaneously, tau hyperphosphorylation has been linked to reduced retrograde transport of neurotrophins from axon terminals to dendrites, further compromising neuronal survival in the AD brain (Lindsay, 1996; Reddy, 2013; Sayas and Ávila, 2021). Conversely, diminished neurotrophin signalling increases susceptibility to Aβ-induced toxicity and promotes tau hyperphosphorylation through reduced inhibition of GSK-3β and other tau kinases (Pollack et al., 2002; Skaper and Walsh, 1998). This bidirectional relationship creates a detrimental environment, leading to deficits in neurotrophin signalling and increased protein aggregation, which further impair neurotrophin function and hasten neurodegeneration. Small molecule neurotrophin mimetics offer several advantages over native proteins. Additionally, small molecules can be optimized for specific pharmacokinetic and pharmacodynamic properties through medicinal chemistry approaches, allowing for enhanced target specificity and reduced off-target effects (Skaper, 2008; Skaper, 2011; Haller et al., 2007; Malakar et al., 2025). Compounds like Amitriptyline have shown promise in restoring neuroprotection, enhancing synaptic function, and improving cognitive performance. Although Amitriptyline has been reported as a dual TrkA/TrkB agonist, its therapeutic role in Alzheimer’s disease remains unestablished beyond *in-vitro* binding studies. The clinical utility of amitriptyline is limited by its severe hepatic and renal toxicity and its ability to disrupt neurite development, leading to neuronal cell death (Thompson et al., 2025; Malakar et al., 2026). To bridge this gap, we developed an Amitriptyline-based pharmacophore model to design novel drug candidates using *in silico* approaches. The designed compounds exhibited interaction patterns similar to Amitriptyline but showed stronger binding affinity toward the TrkA LRR domain, which drives TrkA/TrkB heterodimerization and dual activation. Some compounds also bound directly to the TrkB LRR domain, further supporting potential dual agonist activity. Notably, Amitriptyline itself interacts with the TrkB extracellular domain (K_d_ ∼14 μM) but not TrkC (Kim et al., 2024).

Compounds with favourable TrkB binding energies were retained, while those with higher TrkA affinity were prioritised for MD simulations. DFT analysis revealed that a moderate HOMO–LUMO gap provided an optimal balance of stability and reactivity for effective protein interactions. Among the designed candidates, Chembl35741, a quinoline analogue, emerged as the most promising. Chembl35741 demonstrated favourable electronic characteristics, stable binding (RMSD ∼3.0–3.5 Å), moderate ligand mobility (3–7 Å), and strong, persistent interactions with key residues (*Asp57, His60, Arg85, Asp86*). In contrast, Amitriptyline and CHEMBL51183 showed higher RMSD values and weaker stability, while CHEMBL4864026 displayed only moderate performance.

Further optimisation of Chembl35741 was carried out using Ligand Designer, given its favourable binding affinity and structural stability within the TrkA LRR binding domain. Comparative analysis of the 300 ns MD simulations indicates that OP-1 exhibits the highest binding stability toward TrkA, as evidenced by rapid RMSD equilibration, minimal fluctuations, and persistent key interactions throughout the trajectory. OP-2 shows slightly greater conformational deviation during the early phase of the simulation; however, the system stabilizes after ∼100 ns and remains equilibrated until 300 ns, suggesting reliable and sustained binding, albeit marginally less stable than OP-1. In contrast, OP-3 displays comparatively higher RMSD fluctuations, reflecting increased flexibility and dynamic movement within the binding pocket. Despite this deviation, OP-3 maintains continuous ligand occupancy without dissociation and preserves critical protein–ligand interactions, confirming stable binding. Overall, all three compounds remain stably accommodated within the TrkA binding site, supporting their suitability for synthesis. Among them, OP-1 and OP-2 demonstrate superior stability, while OP-3 may benefit from further structural optimization to enhance binding rigidity in future studies.

The newly designed quinoline derivatives were subsequently evaluated through an integrated *in silico* drug discovery pipeline to identify potential dual agonists for TrkA and TrkB. The optimised quinoline (OP-1 to OP-6) scaffold exhibited favourable ADMET properties were interpreted in the context of CNS drug-likeness criteria. Compounds demonstrating predicted BBB permeability, moderate lipophilicity (optimal LogP range), molecular weight within acceptable CNS limits, and topological polar surface area (TPSA) below ∼90 Å^2^ were considered favorable for brain penetration. P-glycoprotein (P-gp) substrate status was evaluated to assess potential efflux liability, as strong P-gp substrates may exhibit reduced CNS exposure. Toxicity predictions, including hepatotoxicity, cardiotoxicity (hERG liability), and mutagenicity, were critically examined to evaluate safety risk. Overall, the prioritized compounds exhibited ADMET profiles consistent with potential CNS-active agents; however, experimental pharmacokinetic validation remains necessary. High docking affinity, and an appropriate HOMO–LUMO energy gap as confirmed by DFT analysis and stable binding interactions with key amino acid residues within the TrkA leucine-rich domain, including *Ala55, Asp57, Arg85, Arg86, Glu92, Gly44, Cys40*, and *Asn202* (Thompson et al., 2025). These residues are notably associated with the dual agonistic activity of amitriptyline, thereby reinforcing the evidence for potential TrkA/TrkB dual agonism of the designed compound. It is important to emphasize that predicted binding within the LRR domains of TrkA and TrkB does not inherently establish functional agonism. Although docking and MD simulations indicate favorable receptor engagement, agonistic activity requires experimental validation through receptor phosphorylation, ERK/AKT signalling activation, and functional neuroprotection assays. Furthermore, the scaffold exhibits favorable synthetic feasibility, supporting its suitability for subsequent *in-vitro* and *in-vivo* validation. Compared to prior Trk-targeted computational studies, this work offers improved methodological validation, statistical rigor, and dual-receptor binding assessment. Although the findings are computational, the integrated structural, energetic, dynamic, and electronic analyses provide a more comprehensive characterisation of candidate molecules and establish a foundation for future experimental validation in Alzheimer’s therapeutics.

## 5 Study limitations

Despite the comprehensive computational workflow employed in this study, it is important to acknowledge that the findings are based entirely on *in silico* predictions. No *in-vitro* binding, receptor phosphorylation, or functional signalling assays were performed to experimentally validate dual TrkA/TrkB agonism. While molecular docking, MM/GBSA, and long-timescale MD simulations provide strong evidence for stable ligand–receptor interactions, these methods cannot confirm receptor activation or downstream biological responses. Therefore, the identified compounds should be considered as computationally predicted candidates rather than confirmed dual agonists.

## 6 Conclusion and future perspectives

Amitriptyline directly engages LRR domain of TrkA to drive Trk heterodimerization, challenging the long-held view that neurotrophin signalling specificity is dictated solely by protein-ligand complex interactions. This previously unrecognised mode of receptor tyrosine kinase activation provides a conceptual and structural framework for the rational design of small molecules that co-target multiple neurotrophin receptors. Ig2 domain mediates canonical neurotrophin binding, while the LRR domain drives receptor heterodimerization. Quinoline analogues support the feasibility of targeting LRR-mediated heterodimerization through promising *in silico* results. The designed quinoline analogues demonstrated robust multi-parametric computational assessments supporting their potential as putative dual TrkA/TrkB binders. However, these findings remain predictive in nature and require rigorous experimental validation to confirm receptor activation and downstream signalling effects. Future work should focus on experimental validation through TrkA/TrkB protein-binding studies and evaluation of the neuroprotective potential in amyloid-β-intoxicated primary neuronal cultures to confirm the predicted dual agonistic activity of the identified quinoline analogues.

## Data Availability

The original contributions presented in the study are included in the article/Supplementary Material, further inquiries can be directed to the corresponding author.
