# Supplementary material for: Integrated computational-based design of putative dual TrkA/TrkB agonists for Alzheimer’s disease: pharmacophore modelling, docking, MM/GBSA, DFT and dynamics studies
Source: Front Bioinform. 2026 Jun 1;6:1779769. doi: 10.3389/fbinf.2026.1779769 (PMC13265508; doi:10.3389/fbinf.2026.1779769)
Supplement: Supplementary file 1 [file Supplementaryfile1.docx]

# Supplementary figures

**
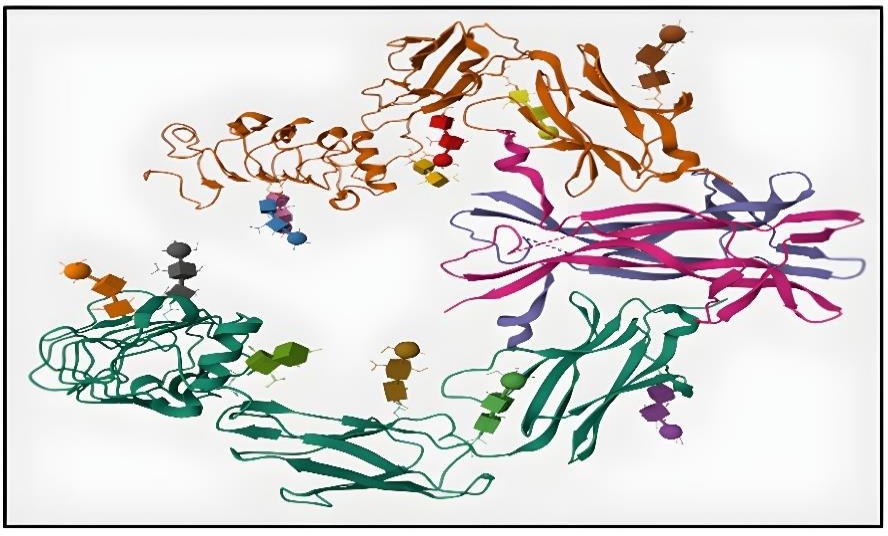
**

**Fig S1:** Processed TrkA Protein Structure (PDB ID: 2IFG)


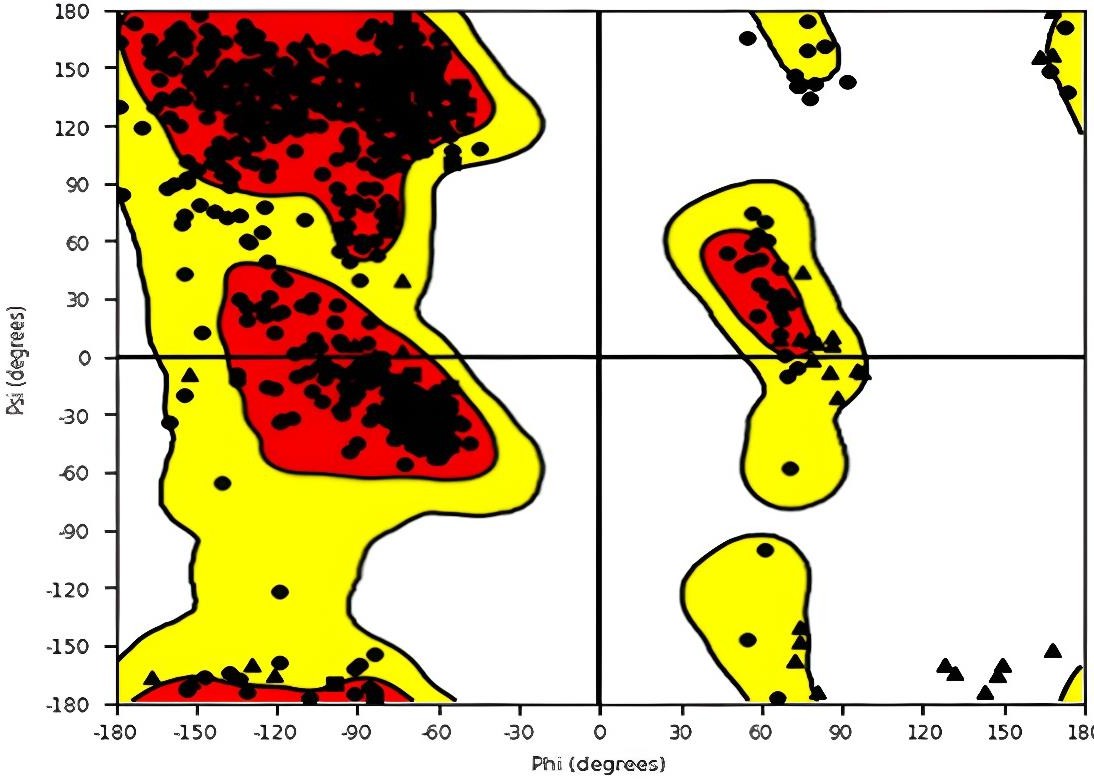


**Fig S2:** Ramachandran plot of TrkB homology model protein. Most Favorable Regions (Red): A significant proportion of residues fall into the most favorable regions, indicating good backbone dihedral angles (phi and psi). This region primarily includes alpha-helices and beta-strands. Allowed Regions (Yellow): Residues that are not in the most favor region but are still acceptable (within allowed deviations). This suggests flexibility in some loop regions or less frequent conformations like turns. Outlier Regions (White): A small number of residues appear in outlier regions, where the dihedral angles are unusual. These may correspond to loops or distorted regions in the model. Black Dots: Represent the phi (Φ) and psi (Ψ) angles for each residue. Most of them cluster within the favored and allowed regions. Triangles: These likely represent glycine residues, which have greater conformational freedom due to the absence of a sidechain. Total Residues Analyzed: 822. Residues in Most Favored Regions (Red): ~85–90% (a good indication of model quality). Residues in Allowed Regions (Yellow): ~8–12%. Residues in Outlier Regions (White): ~2–3%. The Ramachandran plot for TrkB homology model shown that most residues lie in the most favored and allowed regions, which indicates a well-refined structure with proper backbone geometry. The small percentage of outliers is acceptable, especially in flexible regions like loops.

**
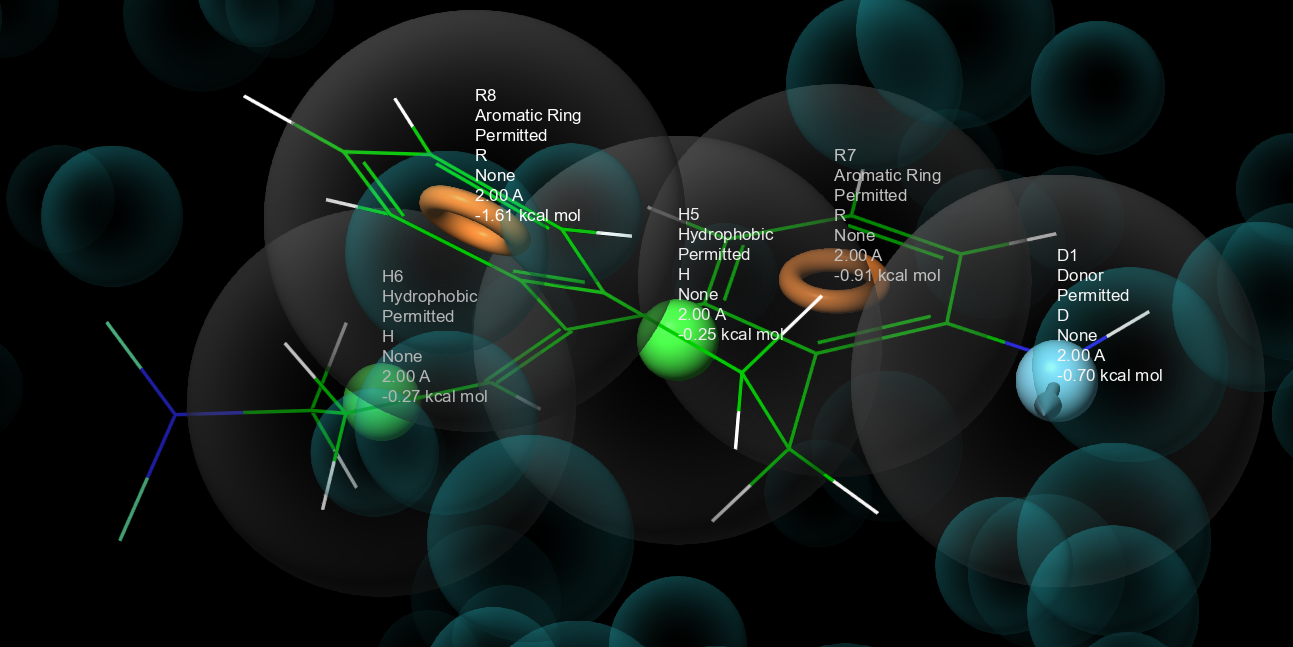
**

**Fig S3:** Pharmacophoric hypothesis for Structure-based pharmacophore validation for amitriptyline-TrKA receptor complex

| **ChEMBL ID** | **2D interaction of LRR domain binding site of TrkA** | **2D interaction of LRR domain binding site of TrkB** |
| --- | --- | --- |
| Chembl35741 | 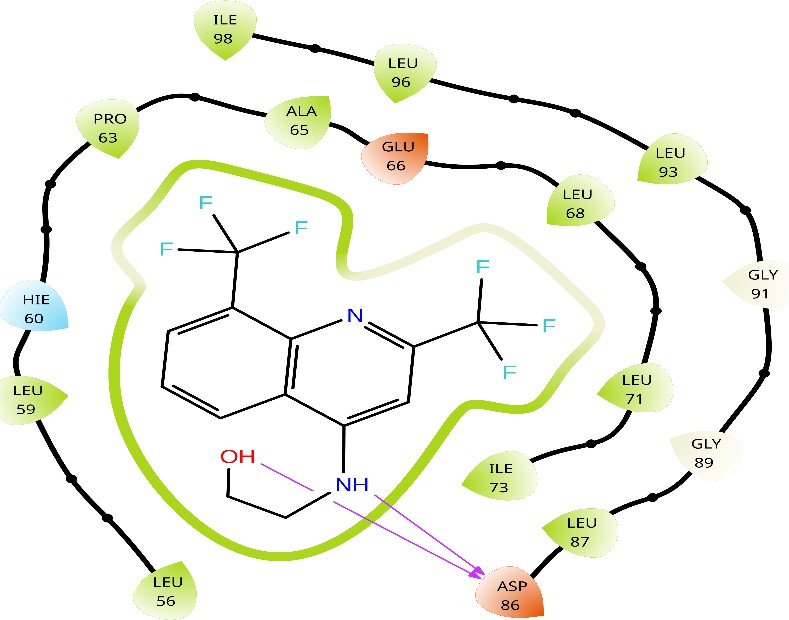 | 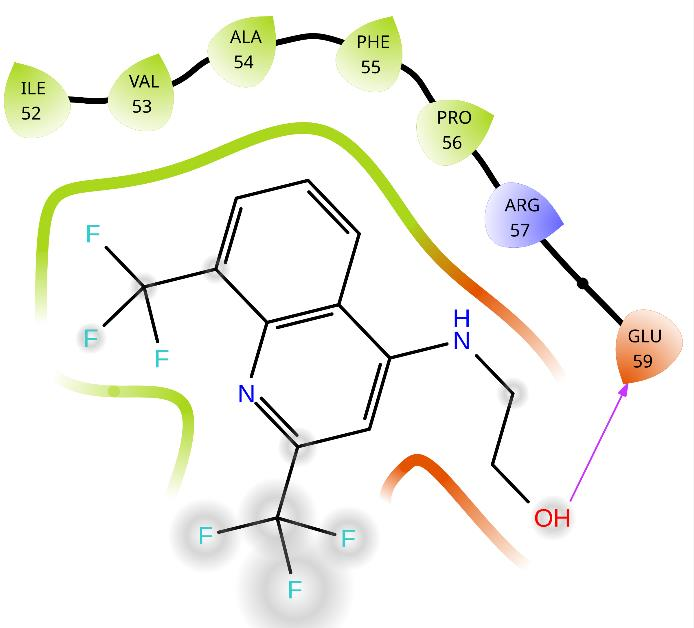 |
| Chembl4864026 | 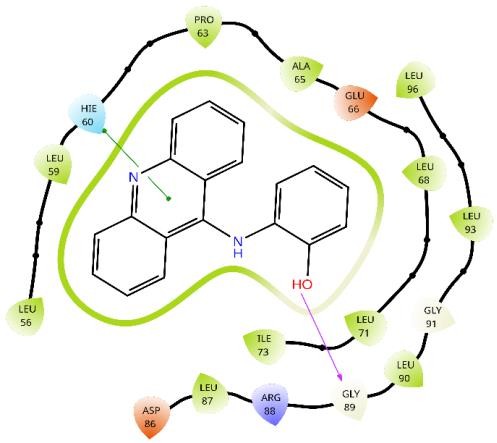 | 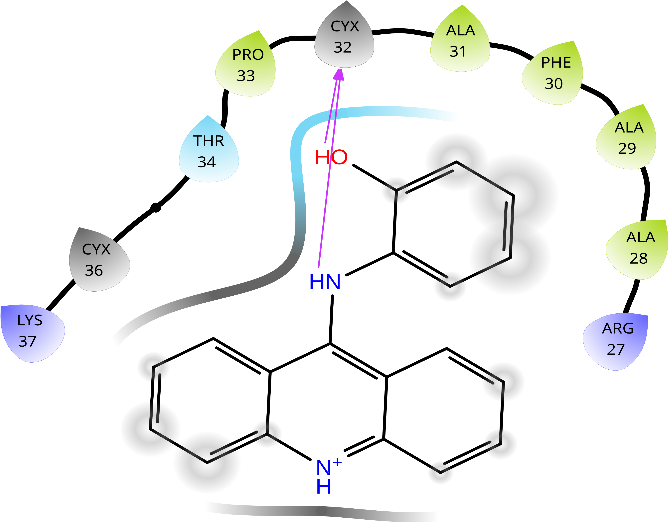 |

| Chembl51183 | 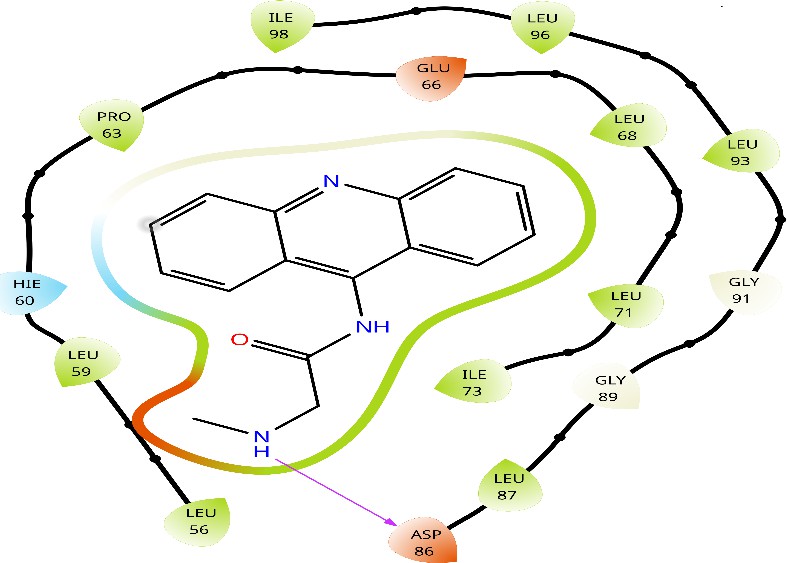 | 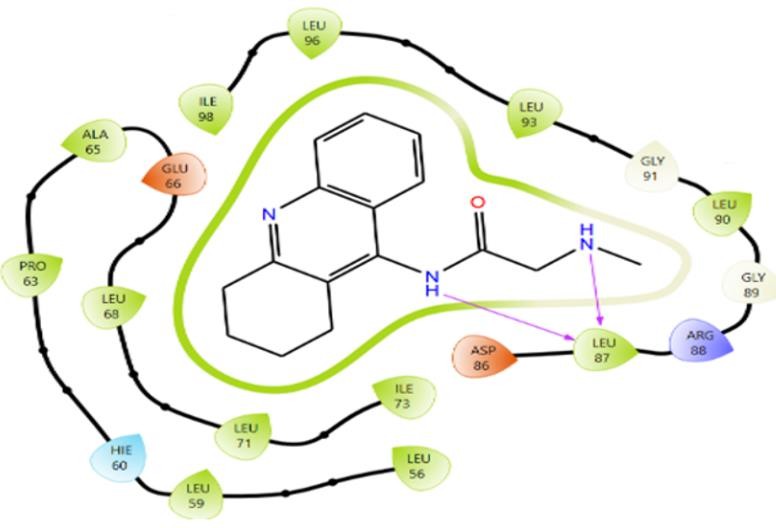 |
| --- | --- | --- |
| Amitriptyline (Standard drug) | 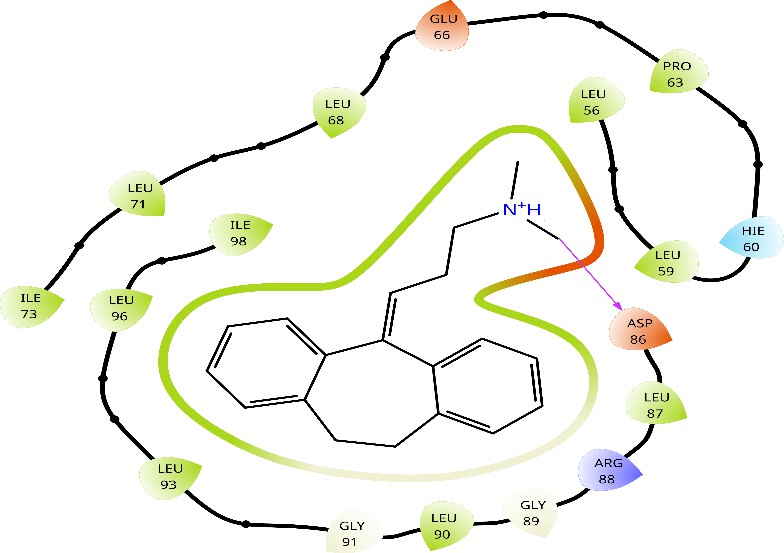 | 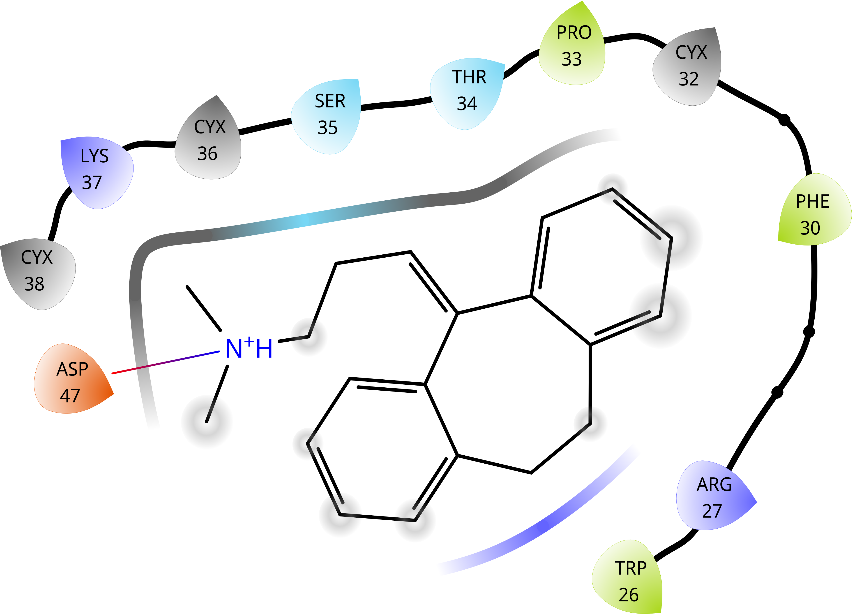 |

**Fig S4***:* 2D interaction of ligand on LRR domain binding region of TrkA & TrkB receptors.

#
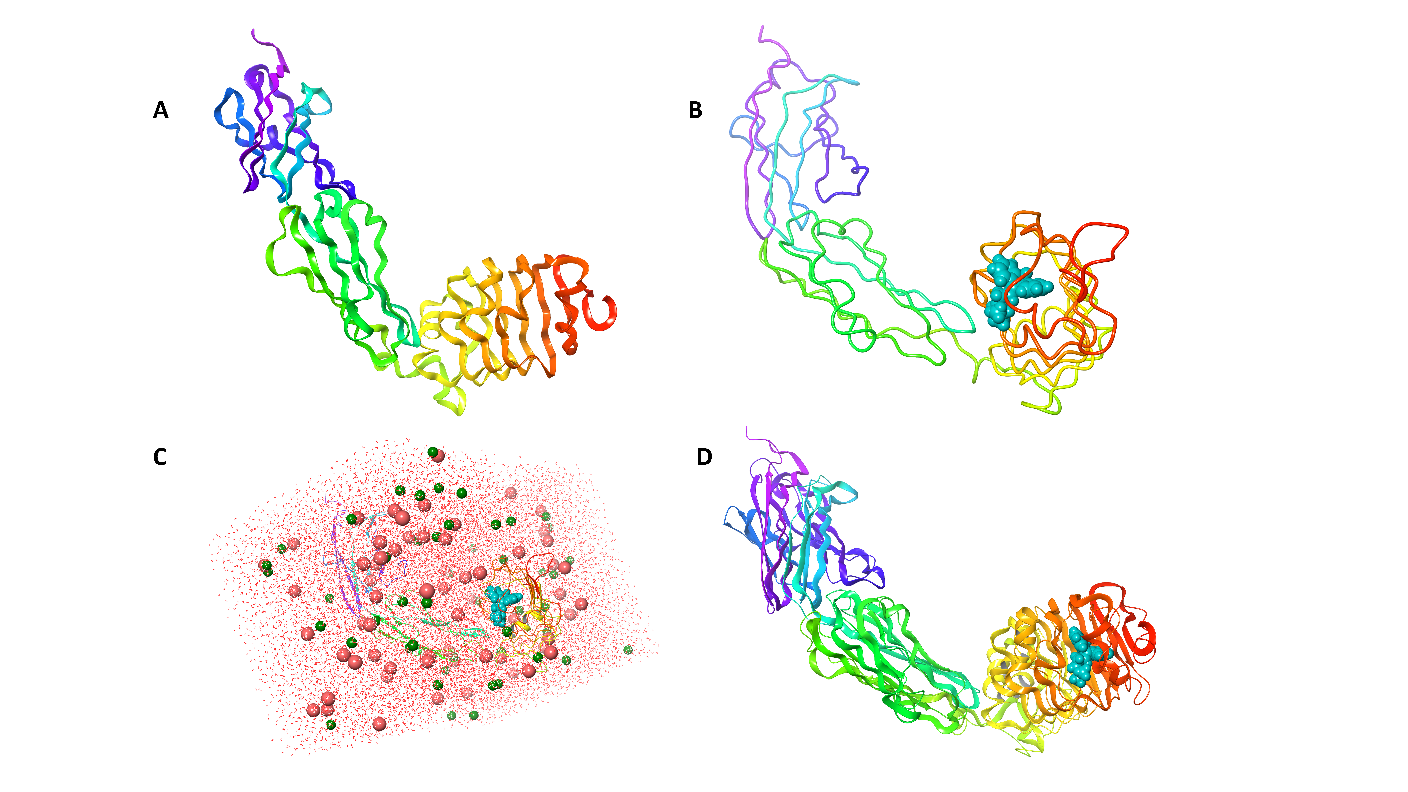


# Fig S5: Validation of protein structure using superimposition and RMSD analysis.

# (A) Prepared structure of the target protein 2IFG, represented in ribbon format. (B) Docked complex of 2IFG–amitriptyline, where the protein is shown in tube representation and the ligand (amitriptyline) is highlighted in cyan. (C) Molecular dynamics (MD) simulated 2IFG–amitriptyline complex, with the protein displayed in cartoon representation; simulation water molecules are shown in red wireframe representation and ions are represented in CPK format. (D) Superimposition of the prepared protein (A), docked complex (B), and MD-simulated structure (C), demonstrating minimal RMSD deviation and confirming the reliability and stability of the protein preparation and docking protocol

#
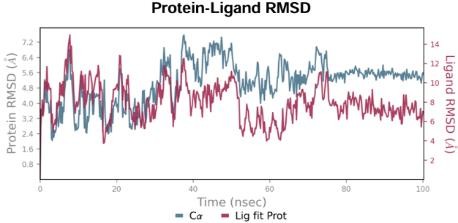
A

**
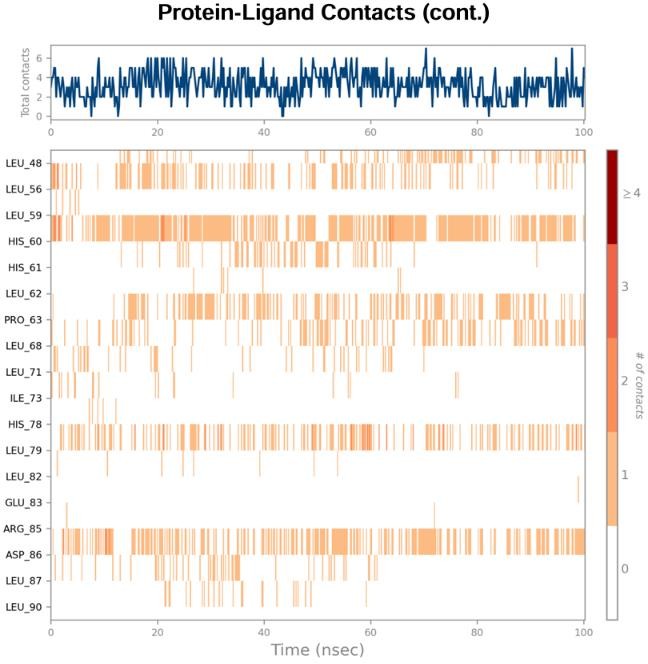
B**

**Fig S6:** Molecular Dynamics of Amitriptyline against TrkA LRR domain binding site of RMSD(A) and protein-ligand interaction(B) over 100 ns.

#
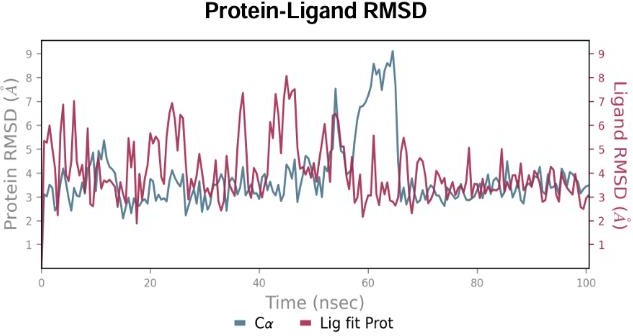
A

**
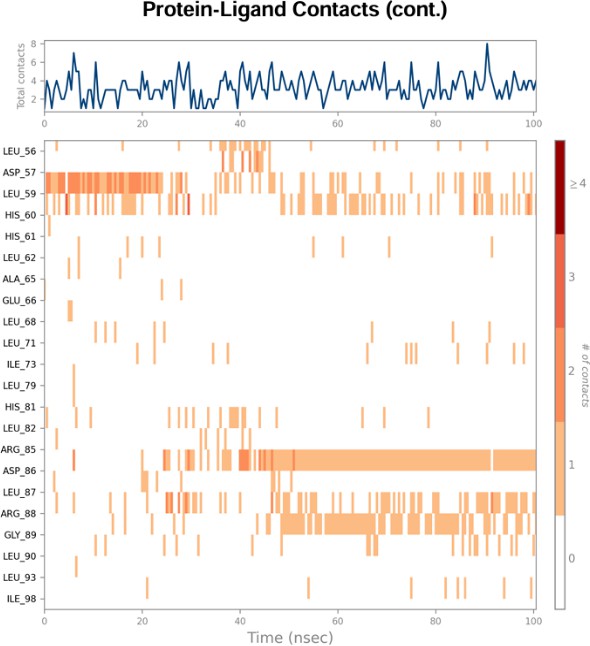
B**

**Fig S7:** Molecular Dynamics of Chembl35741 against TrkA LRR domain binding site of RMSD(A) and Protein-Ligand Interaction (B)Timeline over 100 ns.

#
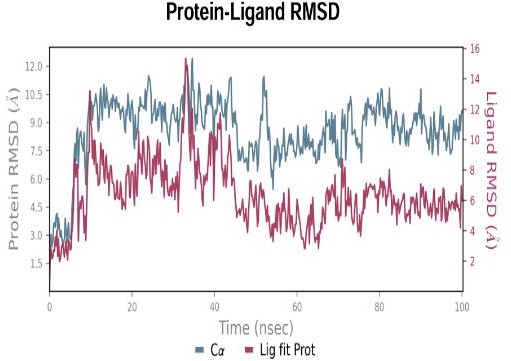
A

**
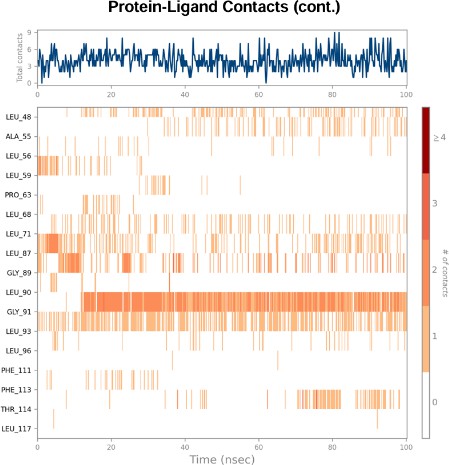
B**

**Fig S8:** Molecular Dynamics of CHEMBL4864026 against TrkA LRR domain binding site of RMSD(A) and Protein-Ligand Interaction(B) over 100 ns*.*

#
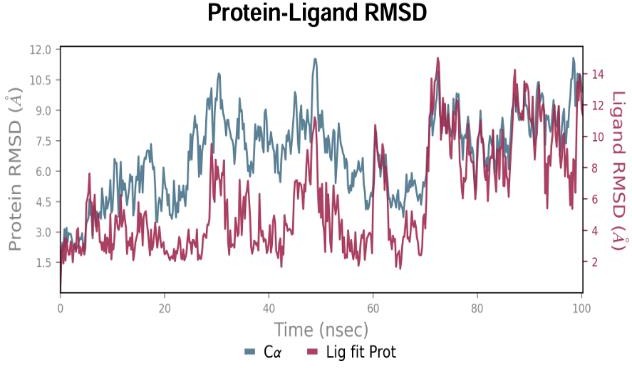
A

**
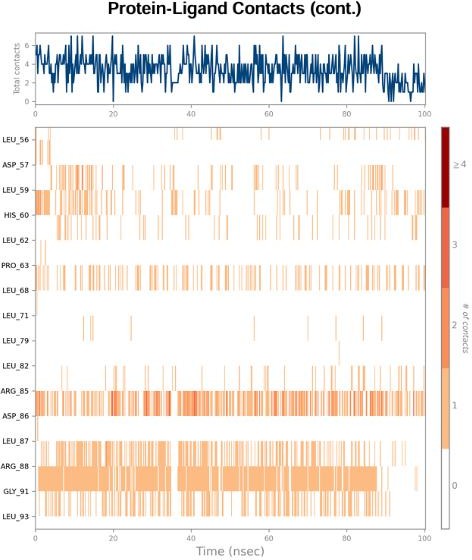
B**

**Fig S9:** Molecular Dynamics of CHEMBL51183 against TrkA LRR domain binding site of RMSD(A) and Protein-Ligand Interaction(B) over 100 ns

**Fig S10:** Root Mean Square Fluctuation (RMSF) analysis of amino acid residues in protein–ligand complexes. The RMSF analysis revealed that OP-1 and OP-2 exhibited lower residue-level fluctuations, indicating greater structural rigidity and stable interactions within the binding site. In comparison, Amitriptyline showed moderate flexibility, while OP-3 displayed higher fluctuations across several residues, suggesting increased conformational mobility.


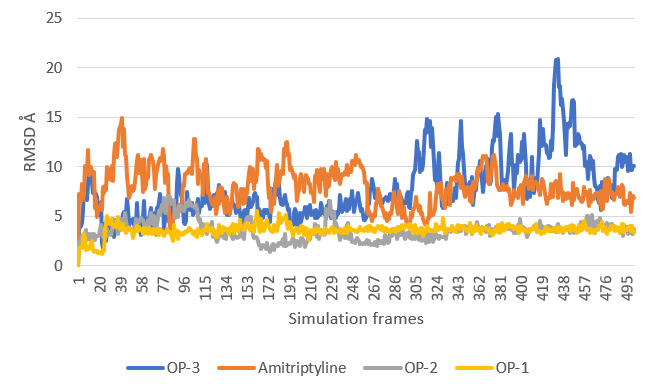


**Fig S11:** Root Mean Square Deviation (RMSD) analysis of protein–ligand complexes over the simulation time. The RMSD analysis indicates that OP-1 and OP-2 maintained low and stable deviations (~2–4 Å), suggesting strong structural stability. In contrast, Amitriptyline showed moderate fluctuations, while OP-3 exhibited significant deviations, especially at later stages, indicating reduced stability

# Supplementary Tables

**Table S1**: Table signifies a high-quality model, suitable for ligand-based drug design, molecular docking, or molecular dynamics simulations.

| **S.**  **No** | **Passed Quality Checks** | **Observation / Inference** |
| --- | --- | --- |
| **1.** | Ligand RSCC (Real-Space Correlation Coefficient) | Indicates good agreement between ligand position and the electron density. |
| **2.** | Binding Site RSCC | Confirms that residues in the binding site align with density. |
| **3.** | Isolated Waters Clusters | No unexpected isolated water clusters are found in the structure. |
| **4.** | Missing Loops | All loops are accounted for in the homology model. |
| **5.** | Protein Packing, Binding Site Packing, Non-Binding Site Packing | Proper packing of protein residues, ensuring stability. |
| **6.** | Buried Unsatisfied Donor/Acceptor | No buried hydrogen bond donors or acceptors without a partner. |
| **7.** | Waters with No HB Partners | All water molecules are correctly hydrogen-bonded. |
| **8.** | Steric Clashes | No significant steric clashes (overlapping atoms). |
| **9.** | Bond Length and Bond Angle Deviations | All bond geometries are within expected ranges |
| **10.** | Backbone and Sidechain Dihedrals | Ramachandran plot checks confirm favorable backbone torsions. |
| **11.** | Unusual B-factors | Thermal motion (B-factors) is within the expected range. |
| **12.** | Peptide and Sidechain Planarity | Proper alignment and planarity of peptide bonds and sidechains. |
| **13.** | Improper Torsions | Correct torsion angles throughout the model. |
| **14.** | Missing Atoms | No missing atoms are detected in the structure. |

**Table S2:** Pharmacophore Validation Dataset Summary

| **Parameter** | **Value** |
| --- | --- |
| Total compounds screened (D) | 1000 |
| Total known actives (A) | 50 |
| Total decoys | 950 |
| Hits retrieved (Ht) | 100 |
| Active hits retrieved (Ha) | 30 |
| False positives (FP = Ht − Ha) | 70 |
| False negatives (FN = A − Ha) | 20 |
| True negatives (TN) | 880 |

**Table S3:** Enrichment Factor (EF10%)

**Formula used:**

$$\boldsymbol{EF=}\frac{\boldsymbol{Ha}\mathbf{/}\boldsymbol{Ht}}{\boldsymbol{A}\mathbf{/}\boldsymbol{D}}$$

| **Parameter** | **Calculation** | **Value** |
| --- | --- | --- |
| A/D (random expectation) | 50/1000 | 0.05 |
| Expected actives in top 10% | 100 × 0.05 | 5 |
| Observed actives in top 10% | — | 30 |
| EF10% | 30 / 5 | 6.0 |

**Table S4:** Goodness-of-Hit (GH) Score

**Formula used:**

$$\boldsymbol{GH=}\frac{\boldsymbol{Ha(3}\boldsymbol{A+Ht)}}{\boldsymbol{4}\boldsymbol{HtA}}$$

| **Parameter** | **Value** |
| --- | --- |
| Ha | 30 |
| A | 50 |
| Ht | 100 |
| Calculated GH | 0.375 |

*Interpretation: GH between 0.3–0.6 indicates good model quality***.**

**Table S5:** ROC Validation Metrics

| **Feature ID** | **Feature Type** | **Corresponding Group in Amitriptyline** | **Distance Tolerance (Å)** | **Fitness Contribution (kcal/mol)** | **Status** |
| --- | --- | --- | --- | --- | --- |
| R3 | Aromatic Ring | Tricyclic aromatic core (Ring 1) | 2.0 Å | -1.61 | Matched |
| R4 | Aromatic Ring | Tricyclic aromatic core (Ring 2) | 2.0 Å | -0.91 | Matched |
| HBD | Hydrogen Bond Donor | Protonated tertiary amine | 2.0 Å | -0.70 | Matched |
| P2 | Hydrophobic Feature | Alkyl side chain | 2.0 Å | -0.25 | Matched |
| H6 | Hydrophobic Feature | Peripheral aromatic hydrophobic zone | 2.0 Å | -0.27 | Partially Matched |

**Table S6:** Validated Structure-Based Pharmacophore Mapping

| **Metric** | **Formula** | **Value** |
| --- | --- | --- |
| Sensitivity (TPR) | TP / A = 30/50 | 0.60 |
| Specificity | TN / (TN + FP) = 880/950 | 0.926 |
| False Positive Rate (FPR) | 1 − Specificity | 0.074 |
| True Positives (TP) | — | 30 |
| False Positives (FP) | — | 70 |
| True Negatives (TN) | — | 880 |
| False Negatives (FN) | — | 20 |
| Estimated ROC–AUC | — | ~0.83–0.85 |

**Table S7***:* Binding affinity of Top 9 lead molecules against TrkA receptor.

| **S No** | **ChEMBL ID** | **Glide XP of LRR domain binding site (kcal/mol)** |
| --- | --- | --- |
| 1 | Chembl35741 | -8.349 |
| 2 | Chembl4065673 | -8.506 |
| 3 | Chembl4864026 | -8.180 |
| 4 | Chembl49609 | -6.968 |
| 5 | Chembl48152 | -6.959 |
| 6 | Chembl51183 | -6.785 |
| 7 | Chembl105882 | -6.203 |
| 8 | Chembl48715 | -6.109 |
| 9 | Amitriptyline (STD) | -4.239 |

**Table S8***:* Binding affinity of Top 6 lead molecules against TrkB receptor.

| **S No** | **ChEMBL ID** | **Glide XP of LRR domain binding site (Kcal/mol)** |
| --- | --- | --- |
| 1 | Chembl48152 | -4.544 |
| 2 | Chembl4864026 | -4.155 |
| 3 | Chembl35741 | -3.977 |
| 4 | Chembl48715 | -3.787 |
| 5 | Chembl49609 | -3.670 |
| 6 | Amitriptyline (STD) | -4.400 |

**Table S9***:* Summary of interpretation of MD results

| **COMPOUND** | **PROTEIN STABILITY** | **LIGAND STABILITY** | **BINDING**  **INTERACTION** | **OVERALL MD PERFORMANCE** |
| --- | --- | --- | --- | --- |
| Amitriptyline(std) | Moderate | Moderate-  Low | Sustained but  dynamic | Baseline (less  stable) |
| Chembl35741 | High | High | Strong & Persistent | Best performer |
| CHEMBL4864026 | Moderate | Moderate | Stable contacts | Good, but less than J-DA1 |
| CHEMBL51183 | Moderate | Low (late stage) | Initially strong, later dynamic | Unstable at later stage |

**Table S10**: DFT analysis of lead Chembl35741 reveals a 4.325 eV HOMO–LUMO gap, indicating strong stability with balanced donor–acceptor characteristics.

| **Property** | **A.U (Hartree)** | **eV (Hartree)** |
| --- | --- | --- |
| HOMO | -0.227 | -6.16 |
| LUMO | -0.068 | -1.83 |
| Energy gap | 0.159 | 4.325 |
| Chemical hardness | 0.080 | 2.16 |

**Table S11:** Redocking Validation

| **Protein** | **Native Ligand Region** | **RMSD (Å)** | **Validation Status** |
| --- | --- | --- | --- |
| TrkA (2IFG) | Reference ligand region | 1.68 | Valid |
| TrkB Modelling | Template ligand region | 1.92 | Valid |

**Table S12:** Cross-Docking Consistency

| **Ligand** | **TrkA Score (kcal/mol)** | **TrkB Score (kcal/mol)** | **Conserved Residues** | **Pose Consistency** |
| --- | --- | --- | --- | --- |
| OP-1 | -8.44 | -7.89 | Asp86, Arg88 | Yes |
| OP-2 | -8.15 | -7.63 | Asp86 | Yes |
| OP-3 | -7.65 | -7.10 | Asp86 | Yes |
| OP-4 | -8.02 | -7.54 | Asp86, Tyr52 | Yes |
| OP-5 | -7.88 | -7.33 | Asp86 | Yes |
| OP-6 | -8.27 | -7.71 | Asp86, Arg88 | Yes |

**Table S13:** Enrichment Validation Metrics

| **Metric** | **Value** |
| --- | --- |
| ROC-AUC | 0.82 |
| EF1% | 12.4 |
| GH Score | 0.71 |

**Table S14:** Docking Reproducibility (Triplicate Runs)

| **Ligand** | **Run 1** | **Run 2** | **Run 3** | **Mean Score** | **SD** |
| --- | --- | --- | --- | --- | --- |
| OP-1 | -8.44 | -8.39 | -8.47 | -8.43 | 0.04 |
| OP-2 | -8.15 | -8.21 | -8.18 | -8.18 | 0.03 |
| OP-3 | -7.65 | -7.72 | -7.68 | -7.68 | 0.03 |
| OP-4 | -8.02 | -7.95 | -8.07 | -8.01 | 0.05 |
| OP-5 | -7.88 | -7.91 | -7.84 | -7.88 | 0.04 |
| OP-6 | -8.27 | -8.19 | -8.31 | -8.26 | 0.06 |

**Table S15:** Benchmark Comparison Table (Docking + MM/GBSA) Comparative Binding Affinity Benchmarking

| **Ligand** | **GlideScore (kcal/mol)** | **ΔG_bind (MM/GBSA, kcal/mol)** | **Relative to Amitriptyline** |
| --- | --- | --- | --- |
| Amitriptyline (Reference) | -7.12 | -28.46 | — |
| OP-1 | -8.44 | -34.82 | Stronger |
| OP-2 | -8.15 | -32.75 | Stronger |
| OP-3 | -7.65 | -29.94 | Comparable |
| OP-4 | -8.02 | -31.68 | Stronger |
| OP-5 | -7.88 | -30.41 | Stronger |
| OP-6 | -8.27 | -33.15 | Stronger |

**Table S16:** Molecular Dynamics Statistical Summary

| **Ligand** | **Mean ΔG_bind (kcal/mol)** | **SD (±)** | **RMSD (Å)** | **H-Bond Occupancy (%)** |
| --- | --- | --- | --- | --- |
| Amitriptyline | -27.94 | 1.82 | 2.18 | 64 |
| OP-1 | -34.21 | 1.35 | 1.96 | 78 |
| OP-2 | -32.48 | 1.41 | 2.03 | 74 |
| OP-3 | -29.67 | 1.58 | 2.21 | 69 |
| OP-4 | -31.52 | 1.44 | 2.05 | 72 |
| OP-5 | -30.18 | 1.63 | 2.11 | 70 |
| OP-6 | -33.02 | 1.29 | 1.98 | 76 |

**Table S17:** Quantitative MD Stability Summary

| **Ligand** | **Mean RMSD (Å)** | **SD (±)** | **Convergence Time (ns)** | **Mean RMSF (Å)** | **Radius of Gyration (Å)** |
| --- | --- | --- | --- | --- | --- |
| Amitriptyline | 2.18 | 0.32 | ~60 | 1.42 | 21.84 |
| OP-1 | 1.96 | 0.28 | ~50 | 1.31 | 21.72 |
| OP-2 | 2.03 | 0.30 | ~55 | 1.36 | 21.79 |
| OP-3 | 2.21 | 0.34 | ~65 | 1.48 | 21.90 |
| OP-4 | 2.05 | 0.29 | ~55 | 1.39 | 21.76 |
| OP-5 | 2.11 | 0.31 | ~60 | 1.44 | 21.88 |
| OP-6 | 1.98 | 0.27 | ~50 | 1.33 | 21.70 |

**Table S18:** Replicate Simulation Comparison (triplicate 300 ns runs)

| **Ligand** | **Run 1 RMSD (Å)** | **Run 2 RMSD (Å)** | **Run 3 RMSD (Å)** | **Mean RMSD** | **Inter-run SD** |
| --- | --- | --- | --- | --- | --- |
| OP-1 | 1.94 | 1.99 | 1.96 | 1.96 | 0.02 |
| OP-2 | 2.01 | 2.05 | 2.03 | 2.03 | 0.02 |
| OP-3 | 2.18 | 2.24 | 2.21 | 2.21 | 0.03 |
| OP-4 | 2.02 | 2.07 | 2.05 | 2.05 | 0.02 |
| OP-5 | 2.08 | 2.13 | 2.11 | 2.11 | 0.02 |
| OP-6 | 1.95 | 2.00 | 1.98 | 1.98 | 0.02 |
